# HER2-Specific Peptide (LTVSPWY) and Antibody (Herceptin) Targeted Core Cross-Linked Micelles for Breast Cancer: A Comparative Study

**DOI:** 10.3390/pharmaceutics15030733

**Published:** 2023-02-22

**Authors:** Nazende Nur Bayram, Gizem Tuğçe Ulu, Nusaibah Abdulsalam Abdulhadi, Seda Gürdap, İsmail Alper İşoğlu, Yusuf Baran, Sevil Dinçer İşoğlu

**Affiliations:** 1 Department of Bioengineering, Faculty of Life and Natural Sciences, Abdullah Gül University, 38080 Kayseri, Turkey; 2 Molecular Biology and Genetics, Faculty of Science, İzmir Institute of Technology, 35433 İzmir, Turkey

**Keywords:** HER2, human epidermal growth factor receptor 2 targeting, breast cancer, stable micelles, the antitumor effect

## Abstract

This study aims to prepare a novel breast cancer-targeted micelle-based nanocarrier, which is stable in circulation, allowing intracellular drug release, and to investigate its cytotoxicity, apoptosis, and cytostatic effects, in vitro. The shell part of the micelle is composed of zwitterionic sulfobetaine ((N-3-sulfopropyl-N,N-dimethylamonium)ethyl methacrylate), while the core part is formed by another block, consisting of AEMA (2-aminoethyl methacrylamide), DEGMA (di(ethylene glycol) methyl ether methacrylate), and a vinyl-functionalized, acid-sensitive cross-linker. Following this, a targeting agent (peptide (LTVSPWY) and antibody (Herceptin^®^)), in varying amounts, were coupled to the micelles, and they were characterized by ^1^H NMR, FTIR (Fourier-transform infrared spectroscopy), Zetasizer, BCA protein assay, and fluorescence spectrophotometer. The cytotoxic, cytostatic, apoptotic, and genotoxic effects of doxorubicin-loaded micelles were investigated on SKBR-3 (human epidermal growth factor receptor 2 (HER2)-positive) and MCF10-A (HER2-negative). According to the results, peptide-carrying micelles showed a higher targeting efficiency and better cytostatic, apoptotic, and genotoxic activities than antibody-carrying and non-targeted micelles. Also, micelles masked the toxicity of naked DOX on healthy cells. In conclusion, this nanocarrier system has great potential to be used in different drug-targeting strategies, by changing targeting agents and drugs.

## 1. Introduction

In recent years, nanocarriers have been used effectively in cancer treatment due to their remarkable properties, such as accumulation at the tumor site with the EPR (enhanced permeability and retention) effect, being stimulus-sensitive, and an ability to target the tumor site with a specific ligand. Numerous studies are still being carried out to increase the effect of nanoparticles, by adding new properties to nanoparticles [1,2]. Polymeric micelles, one of the nanoparticle types, have been studied comprehensively, due to their ability to increase solubility, reduce drug toxicity, and allow the targeting of tumor areas with specific ligands. Polymeric micelles are formed by self-assembling a diblock copolymer, consisting of hydrophilic and hydrophobic blocks, giving the abovementioned properties. Numerous hydrophilic polymers have been studied as the shell of the micelles, and polyethylene glycol (PEG) is the most widely used, because of its superior biocompatibility and stealth effect against proteins [3,4,5]. However, a recent study reported that PEG-carrying micelles showed an unexpected immunogenic response because of the accelerated blood clearance (ABC) phenomenon, resulting in the rapid removal of nanocarriers and reduced efficacy [6]. Although PEG is still frequently used in nanocarrier structures, potential candidates with similar characteristics and non-immunogenicity have been searched. Recently, micelles containing zwitterions have received much interest, due to their high biocompatibility and non-bioadhesive characteristics [7,8]. Betaine polymers consist of anion and cation groups in the same molecule, that give these zwitterionic polymer properties. In addition, betaine polymers such as polysulfobetaine are characterized by a high biocompatibility rate due to their structure, similar to phosphatidylcholine (PC), located in the cellular membrane [9]. Moreover, betaine polymers are sensitive to several stimuli, such as pH and temperature, as a type of upper critical solution temperature (UCST). Using zwitterionic polymers in the structure of carrier systems has recently been reported for cancer treatment purposes. Fuji et al. prepared betaine-based nanoparticle bearing zwitterionic polymers, and they found that these nanoparticles showed efficient tumor permeability compared to a nonionic PEGMA-based nanoparticle [10]. Studies have also shown that sulfobetaine methacrylate-functionalized nanoparticles improve cancer treatments, due to their long circulation times and similarity to cell membranes that increase uptake by cancer cells [11,12,13,14].

Although micelles containing betaine groups have these superior properties, an early release may be encountered in self-assembly-formed micelles. To prevent the early release, and increase the stability of the micelles, there are several studies in which core cross-linked micelles (CCMs) are synthesized. In the synthesis of cross-linked micelles, acid-sensitive micelles can be obtained by using cross-linkers containing acid-sensitive acetal and ketal groups, thus preventing early release and releasing the drug in the tumor region, which is more acidic than the blood [15].

RAFT polymerization is the most demanded technique for synthesizing different macromolecular architectures, with a large range of monomer systems allowing uniformity in chain length and resulting in a well-defined polymer with a low PDI (polydispersity index). Besides that, self-assembled micelles can be easily cross-linked before, during, and after polymerization, with the micelles’ living group by adding divinyl compounds to the solution [16,17,18,19]. It gives stability to the micellar structure, preventing premature drug release, with smart nanocarrier characteristics. Also, owing to the living radical group in the macromolecular structure, RAFT polymerization gives an opportunity to conjugate biomolecules like peptides and antibodies.

Since passive targeting of nanocarriers is insufficient to reach the desired location, more effective active targeting methods are needed. In this context, ligands recognizing target molecules expressed in large numbers on the surface of tumor cells, are added to the nanocarrier structure with appropriate methods, to achieve results such as directing the drug to the target and reducing the side effects of chemotherapy. Ligands bound to these carriers can be in peptide, antibody, or aptamer structures, which offer different targeting efficiencies [20,21,22]. Despite the absence of a naturally occurring ligand for the HER2 receptor, various artificial ligands such as antibodies, Fab fragments, single-chain variable fragments, affibodies, and peptides have been developed for targeted drug delivery. One of the most well-investigated strategies for targeted drug delivery to the HER2 receptor is the utilization of antibodies that can recognize the HER2 receptor, and conjugate with them nanoscaled systems such as nanoparticles and immunoliposomes. Human epidermal growth factor receptor 2 (HER2) is a family member of the receptor tyrosine kinases (RTKs), responsible for cellular proliferation and tumorigenesis. The overexpression of HER2 is found in approximately 20% of human breast cancers. To inhibit HER2 overexpression and/or amplification, trastuzumab (Herceptin^®^), pertuzumab (Perjeta^®^), or chemotherapeutic agents are used clinically [23,24]. Studies have shown that immunoliposomes conjugated with anti-HER2 antibodies have a prolonged circulation in the bloodstream, and selectively deliver drugs such as doxorubicin, to HER2-positive tumors [25,26,27]. Due to the loss of activity in antibody-based ligand studies, peptides specifically selected for the HER2 receptor region in SKBR3 cells, by the phage display technique, are also recommended as an alternative that would be more advantageous [28,29,30,31,32]. The peptide LTVSPWY, is another ligand that was discovered through a technique called biopanning, which uses a library of peptides to identify binding partners through affinity selection. This peptide has been used to target various receptors, including HER2. It has been used to deliver an antisense oligonucleotide specifically to HER2-positive tumor cells [33,34]. Additionally, it has been used to deliver a pro-apoptotic compound, called alpha-tocopheryl succinate (alpha-TOS), selectively to HER2-overexpressing cancer cells, also it has been conjugated to magnetic nanoparticles for imaging purposes [35,36].

Herein, we aimed to prepare core cross-linked micelles, which targeted HER2-positive breast cancer cells, with pH-sensitivity features, and compare targeting efficiencies of an HER2-specific peptide (LTVSPWY) and Herceptin antibody towards breast cancer cells. To synthesize the shell part of the micelles, firstly a sulfobetaine block, as a macroCTA, was synthesized by RAFT polymerization. Following this step, macroCTA was copolymerised with di(ethylene glycol) methyl ether methacrylate (DEGMA) and aminoethyl methacrylamide (AEMA), and an acid-sensitive cross-linker, to obtain CCMs. For comparison of the targeting efficiencies of peptide and antibody, an HER2-specific peptide (LTVSPWY) and an HER2-specific antibody (Herceptin) were conjugated to the micelles. Then, doxorubicin (DOX) was loaded into the micelles by the incubation method. The cytotoxicity of CCMs and targeted CCMs was investigated using HER2-positive SKBR3 cells and the healthy breast cell line MCF-10A. Moreover, the apoptotic and cytostatic effects of CCMs, HER2 peptide-conjugated CCMs, and HER2 antibody-conjugated CCMs were analyzed on breast cancer cells. In conclusion, these CCMs revealed high potential as a drug delivery system for breast cancer, with improved stability, targeting, and pH sensitivity properties.

## 2. Materials and Methods

### 2.1. Materials

4-Cyano-4-(phenylcarbonothioylthio) pentanoic acid (CTA), 4,4′-Azobis(4-cyanovaleric acid) (ACVA), diethylene glycol methyl ether methacrylate (DEGMA), 2,2-dimethoxypropane, 2-hydroxyethyl methacrylate (HEMA), p-toluenesulfonic acid monohydrate (p-TSA.H_2_O), triethylamine, N-(3-dimethylaminopropyl)-N′-ethylcarbodiimide hydrochloride (EDAC.HCl), N,N-diisopropyethylamine (DIPEA), and 1-hydroxybenzotriazole hydrate (HOBT), were purchased from Sigma-Aldrich, Waltham, MA, USA. N-(3-Sulfopropyl)-N-methacroyloxyethyl-N,N-dimethylammonium betaine (sulfobetaine), 1,4 Dioxane, 2-Propanol, hexane, ethyl acetate, sodium nitrate, and acetonitrile for liquid chromatography, were purchased from Merck, Darmstadt, Germany. N-(2-Aminoethyl) methacrylamide hydrochloride (AEMA) was purchased from Polyscience, Evantson, Ilinois, USA and sodium azide from SERVA, Heidelberg, Germany. Synthetic peptide (LTVSPWY) was purchased from CASLO Laboratory ApS, Lyngby, Denmark with a purity of 95% and a molecular weight of 864.99 g/mol. Pierce™ BCA Protein Assay Kit and Pierce Quantitative Fluorometric Peptide Assay were purchased from Thermo Scientific, Waltham, MA, USA. All other chemicals used were of analytical grade. SKBR-3 (HER2-positive human mammary gland/breast adenocarcinoma) cell line was purchased from ATCC, Manassas, VA, USA (HTB-30). MCF-10A (HER2-negative human mammary gland/breast non-tumorigenic) cell line (ATCC CRL-10317) was kindly provided by Prof. Dr. Ayşe Elif Erson Bensan, Middle East Technical University. MTT formazan powder for cell proliferation (Merck, Darmstadt, Germany), paraformaldehyde (PFA) powder, low-gelling-temperature agarose, and propidium iodide dye were from Sigma-Aldrich, Waltham, MA, USA. The fluorescent dye 4′,6-diamidino-2-phenylindole dihydrochloride (DAPI) was provided by Invitrogen, Waltham, MA, USA. Annexin V/PI dual staining apoptosis assay kit and propidium iodide dye were obtained from Biolegend, San Diego, CA, USA. RNase-A was obtained from Biomatik, Wilmington, DE, USA. Trans-Blot^®^ Turbo TM Transfer System, including TGX Stain-Free TM FastCast TM acrylamide kit (10%), Trans-Blot^®^ Turbo TM RTA Transfer Kit-PVDF, and Clarity TM Western ECL Substrate from BIORAD, Hercules, CA, USA were used for western blotting. The ab32124-Anti-Bcl-2 antibody (E17), ab32503- Anti-Bax antibody (E63), ab9485- Anti-GAPDH antibody (Loading control), and ab205718 Goat Anti-Rabbit IgG (HRP), from Abcam, Cambridge, MA, USA were used for the determination of protein expression.

### 2.2. Methods

#### 2.2.1. Synthesis of Homopolymer and Core Cross-Linked Micelles (CCMs) by RAFT Polymerization

Poly(SBMA) was synthesized with RAFT polymerization to obtain the macroCTA, according to our previous study [37]. Briefly, SBMA (sulfobetaine methacrylate), a chain transfer agent (CTA, 4-cyano-4-(thiobenzoylthio) pentatonic acid), and an initiator (ACVA) were dissolved in 0.5 M aqueous NaCl with pH 7–7.5, with the initial molar ratios of monomer to chain transfer agent to initiator, [M]_0_/[CTA]_0_/[I]_0_ = 65/1/0.2, and the solution was sealed and purged with N_2_ for 30 min. Then, the solution was heated to 70 °C. To analyze the reaction mechanism, if it undergoes RAFT, aliquots were withdrawn with a syringe from the reaction medium at predetermined intervals during polymerization. All the samples were precipitated with cold diethyl ether three times and dried under a vacuum. Conversion of the monomer was calculated gravimetrically. All the samples were analysed with ^1^H-NMR spectroscopy and GPC (Gel Permeation Chromatography). GPC analysis was performed by a TOSOH EcoSEC HLC-8320 GPC/SEC System with an RI Detector, Wyatt miniDAWN Treos-II MALS (Multi-Angle Light Scattering) detector equipped with PSS SUPREMA analytical 100 Å column (8 × 300 mm, 10 μm, PSS Polymer Standards Service Inc., MA, USA) at room temperature. As a mobile phase, 80% aqueous 0.1 M ammonium sulfate −20% acetonitrile with 0.0125% sodium azide was used, with a flow rate of 2.0 mL/min. The injection volume of the filtered (by 0.2 μm PTFE filter) poly(SBMA) solutions was adjusted to 50 mL. For RAFT-functionality estimation, macroCTA’s conversion was kept at a low degree (for 2 h) and the resulting pure polymer was analyzed by ^1^H NMR spectroscopy.

**Scheme 1:**
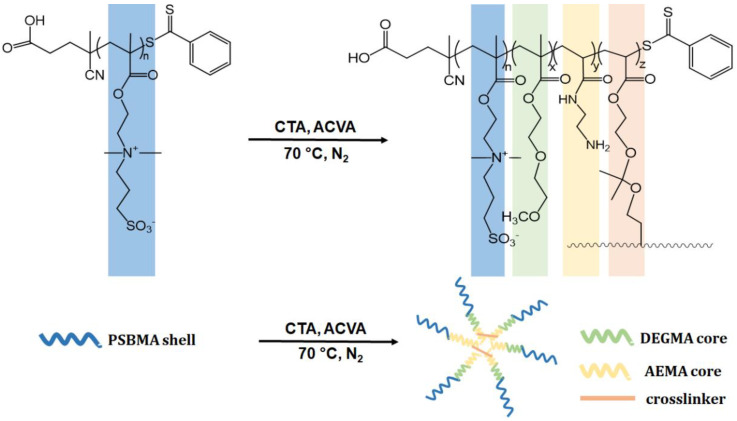
Synthesis of CCMs by RAFT polymerization.

To synthesize the CCMs, we first synthesized an acid-sensitive cross-linker (CL, 2,2-dimethacroyloxy-1-ethoxypropane), according to the literature [38]. Following cross-linker synthesis, we proceeded to synthesize macroCTA, followed by the synthesis of CCMs by RAFT polymerization (Scheme 1), as reported in our previous study. To synthesize the micelle, macroCTA (0.0033 mmol), AEMA (0.26 mmol), DEGMA (0.75 mmol), and CL (5% mmol) were dissolved in water, and the reaction was kept overnight at 70 °C, using ACVA as the initiator. The chemical structures of the purified copolymer micelles were characterized by FTIR and ^1^H-NMR spectroscopies. The sample size and size distribution analyses were determined by dynamic light scattering spectroscopy [37].

#### 2.2.2. Preparation of Targeted CCMs

HER2-specific peptide (LTVSPWY) and antibody (Herceptin) conjugation:

Due to the hydrophobic nature of the peptide, the peptide conjugation reaction was carried out in an organic solvent. For the peptide conjugation to the CCMs’ COOH groups, which come from the RAFT polymerization, 100 mg of CCMs was first dissolved in 3 mL of DMSO/DMF (3:1) mixture. Then, this solution was stirred at 60 °C overnight, and cooled to 25 °C. Following this step, EDC (3.07 mg), HOBT (2.23 mg), DIPEA (4.18 µL), and varying amounts of peptide (13.84, 1.38, 0.55, 0.27, 0.14 mg, LTVSPWY) were added to the solution and stirred at 35 °C for two days. In order to remove unbounded peptide, the solution was dialyzed against water for about 24 h, and then freeze-dried. (Fisherbrand Regenerated Cellulose Dialysis Tubing, MWCO 3500) [39,40,41].

For antibody conjugation to the CCMs’ COOH groups, 100 mg of CCMs was dissolved in 4 mL of PBS containing 0.9% NaCl, stirred at 60 °C overnight, and cooled to room temperature. EDC (3.07 mg), sulfo-NHS (4.4 mg), and varying amounts of Herceptin (60, 30, 15, 5, and 1 mg) were added to this solution and stirred at room temperature for two days. The solution was dialyzed against water (Fisherbrand Regenerated Cellulose Dialysis Tubing, MWCO 3500) for about 24 h. Then, the sample was centrifuged in the tubes, with a 300,000 MWCO membrane, to remove unconjugated Herceptin (Vivaspin, 300,000 MWCO), and the product was lyophilized [42,43]. The products were analyzed by FTIR and ^1^H-NMR spectroscopies to confirm peptide and antibody coupling. A BCA protein assay kit (Pierce BCA Protein Assay, Thermo Scientific, Waltham, MA, USA) was used for the antibody coupling quantification. We estimated the peptide content on the nanocarrier using a fluorescence spectrophotometer at Ex/Em 280/350 nm. Size and charge analyses of the micelles were performed by a Malvern Zetasizer (Malvern). The morphology of the peptide and antibody-coupled CCMs were examined by SEM (scanning electron microscope; Carl Zeiss EVO LS10, NTS, Germany).

#### 2.2.3. Drug Loading Study

The CCMs, peptide-conjugated CCMs, and antibody-conjugated CCMs were dissolved in DMSO. Then, doxorubicin.HCl and TEA (3×DOX.HCl) were added to the solution and stirred overnight, with protection from light. In order to remove unbounded DOX from micelles, these solutions were dialyzed against water (Fisherbrand Regenerated Cellulose Dialysis Tubing, MWCO 3500), and the water solution was changed three times and lyophilized [44,45]. To calculate the amount of doxorubicin, 1 mg of micelles was dissolved in 1 mL of DMSO, and the absorbance of the micelles was measured using UV-Vis spectrophotometry at 496 nm, and EE% and LE% were calculated based on the formula given below [18];

Loading efficiency (%) (LE%) = (Amount of DOX in micelles (mg)/Amount of the micelles (mg)) × 100

Encapsulation efficiency (%) (EE%) = (Amount of DOX in micelles (mg)/Initial amount of DOX (mg)) × 100

The method used for the DOX quantification was validated according to the ICH Guidelines [46].

#### 2.2.4. Drug Release Study

Doxorubicin-loaded micelle solution (2 mL), with a concentration of 1 mg/mL, was placed in a dialysis membrane (Fisherbrand Regenerated Cellulose Dialysis Tubing, 12,000 MWCO). The dialysis membrane was placed into 0.05% SDS, containing acetate buffer (10 mM, 150 mM NaCl, pH 5) or PBS (10 mM, pH 7.4) buffer, and shaken (100 rpm) at 37 °C. At predetermined time intervals, 1 mL of buffer solution was withdrawn and replaced with fresh buffer. DOX release was determined by measuring the absorption (at 496 nm) of the DOX molecule in withdrawn buffers, and the cumulative release plots were obtained using the formula below.

CR (%) = [(100 × ((Vm × CDOX(n)) + (1 mL × Σ CDOX(n − 1)))]/W_0_

According to this, Vm: emission media volume; W_0_ (mg): the amount of drug loaded; CDOX (n): the amount of DOX (mg/mL) in the sample taken from the release medium; CDOX (n − 1): (n − 1). the amount of DOX in the sample taken from the media.

#### 2.2.5. Preparation of Cell Lines

SKBR-3 (HER2-positive breast cancer cells) and MCF-10A (HER2-negative non-tumorigenic breast cells) were used to understand the effect of CCMs. The MCF-10A cell line was used as control. SKBR-3 cells were cultured in Dulbecco’s Modified Eagle Medium (DMEM) high glucose with 10% fetal bovine serum (FBS), 1% L-glutamine, and 1% Pen-Strep. Besides, MCF-10A cells were cultured in Dulbecco’s MEM Nutrient Mix F12 (1:1) with 10% horse serum, 1% L-glutamine, 1% Pen-Strep, 20 ng/mL epidermal growth factor (EGF), 0.5 µg/mL hydrocortisone, 10 µg/mL insulin, and 100 ng/mL cholera toxin.

Note that, in all cytotoxicity analyses, experiments were performed in triplicate and repeated three times under similar conditions, and paired *t*-test was performed for statistical analysis, and *p* < 0.05:*; *p* < 0.01:**; *p* < 0.001:*** were considered significant.

#### 2.2.6. Cell Proliferation

The breast cancer cells (SKBR-3) and healthy cells (MCF-10A) were seeded in 96-well plates for 24 h before treatment. After 24 h, both cell lines were treated with 100 µL of dH_2_O as a blank, different free CCMs with different concentrations. After 48 and 72 h incubation, 10 µL of prepared MTT ((3-(4,5-Dimethylthiazol-2-yl)−2,5- Diphenyltetrazolium Bromide) Sigma-Aldrich) solution (5 mg/mL) was added into each well and incubated with 5% CO_2_ at 37 °C for 4 h. After incubation, formazan dye formation was centrifuged, and then the supernatant was removed. The 100 µL of DMSO was added to each well and mixed thoroughly with the pipette. Next, it was incubated at 37 °C for 10 min, and the dissolved form of formazan dye was read at 570 nm with spectrophotometry (Thermo Electron Corporation Multiskan Spectrum, Waltham, MA, USA). After determination of the cytostatic effects of the free CCMs, all other treatments with DOX-loaded CCMs with different concentrations were performed on the cells.

IC50 (the half maximal inhibitory concentration) values, representing the concentration at which the drug causes 50% inhibition of cellular proliferation, were calculated for the DOX-loaded CCMs in this study by plotting the inhibition of cellular proliferation against the drug concentration. To calculate IC50, we obtained a series of dose-response data (e.g., drug concentrations x1, x2, ..., xn and percentage of growth inhibition y1, y2, ..., yn) and the values of y were in the range of 0–100. We then plotted x-y and fitted the data in a straight line (linear regression), and the IC50 value was calculated using the following formula:

Y = a * X + b,      IC50 = (0.5 − b)/a

Since it was determined as 0.65 μM for SKBR-3 cells and 0.08 μM for MCF-10A in our previous study, we performed the experiment in these concentration ranges for DOX-loaded CCMs in the cell proliferation study [19].

#### 2.2.7. Intracellular Uptake of CCMs

In order to determine the cellular uptake profile of CCMs, both cell lines were treated and incubated with CCMs for 48 h. After incubation, cells were washed and fixed with 3.7% paraformaldehyde (PFA) for 20 min. Then, 1:500 DAPI, in 2 ml 1× PBS solution, was added and incubated for 30 min. Each well’s ten different images were taken with an Alexa−555 for 210.5 ms with Olympus-IX83 fluorescence microscopy. The integrated fluorescence intensity was quantified using the ImageJ software [47,48]. All experiments were tested and analyzed in triplicate, and repeated three times with similar conditions and paired *t*-test was performed for statistical analysis, and *p* < 0.05:*; *p* < 0.01:**; *p* < 0.001:*** were considered significant for 10 different image sections. The error bars represent the standard deviations.

#### 2.2.8. Apoptotic Effects

The apoptotic effect of micelles on SKBR-3 was investigated using an Annexin V/PI double staining apoptosis assay and a JC-1 mitochondrial membrane potential assay kit (Supplementary Figure S7), and determining Bax and Bcl-2 protein expression levels, with Western blotting. The percentage of apoptotic and necrotic cells was determined by Annexin V/PI dual staining apoptosis assay (BioLegend, San Diego, CA, USA). After incubation of the CCMs on cells at 48 h, the cells were centrifuged and dissolved in 200 µL/well Annexin binding buffer. FITCH (2 µL) and/or 2 µL PI were added and incubated at room temperature for 15 min. Finally, apoptotic or necrotic cells were determined using flow cytometry (BD FACS CANTO). Besides, apoptosis-related Bcl-2 and Bax protein expression levels were measured by Western blotting. After incubation of CCMs on cells, cells were lysed within 10 mM Tris-HCl, 1 mM EDTA, and 0.1% Triton-X buffer. The protein amount was measured by using a SMART TM BCA protein assay kit. After this, 30 µg of the protein sample of the cell lysed is separated on SDS-PAGE gel and transferred to the membrane. The target protein was bound and visualized using 1:1000 Bcl-2 and 1:1000 Bax primary antibodies and 1:3000 IgG rabbit secondary antibody. In addition, 1:2500 GAPDH primary antibody was used for loading control. The Bcl-2 and Bax expression levels were quantified by normalization of the GAPDH protein. All of the apoptotic analyses were done in at least three independent experiments.

#### 2.2.9. Cytostatic Effects

The cytostatic effects of CCMs on cells was investigated by flow cytometry-based cell cycle analysis, that was based on the quantification of DNA content in cells. In order to do this, propidium iodide (PI) was used to reveal the distribution of cells in three major phases of the cycle (G1 vs. S vs. G2/M). After incubation of the CCMs, the cells were fixed in ice-cold 70% ethanol overnight, at −20 °C. After fixation, cells were collected and incubated in 1 mL PBS-(0.1%) Triton-X 100 with RNase-A for 30 min. Next, the fixed cells were incubated with 100 µL PI at room temperature for 10 min. Then, the different phases of the cell cycle were determined with flow cytometry. Experiments for the cell cycle analysis were performed in at least three independent experiments.

#### 2.2.10. Genotoxic Effects

The genotoxic effects of DOX-loaded CCMs, peptide, and antibody-conjugated CCMs on SKBR-3 cells were analyzed using a comet assay. After 48 h-incubation-time of DOX-loaded CCMs, peptide, and antibody-conjugated CCMs on cells, the cells were harvested and centrifuged. Before starting the comet assay, slides were precoated with 1% agarose. Next, 1% low-gelling-temperature agarose in 1× PBS was prepared and mixed with the cells. The solution (75 µL) was dropped on the slide, and immediately a coverslip was placed on the dropped gel. The slides were incubated at 4 °C for 5 min. After incubation, the coverslips were removed, and the slides were placed in a lysis buffer solution at 4 °C for 1 h. After the lysis buffer, the slides were incubated with an enzyme buffer at 37 °C for 2 min. After lysis and enzyme digestion, the slides were placed in an agarose tank and filled with an alkaline electrophoresis buffer. Electrophoresis was run at 4 °C and a voltage of 25 volts, for 40 min. After electrophoresis, slides were washed with a neutralization buffer and water. Then, a 10 μg/mL stock solution of propidium iodide was put on each slide and incubated for 20 min. Different images were taken for each slide using 210.5 ms with Olympus-IX83 fluorescence microscopy. Finally, the images were processed by using the TriTek CometScore 2.0 program based on comet/tail formation.

## 3. Results

### 3.1. Synthesis and Characterization of Homopolymers, Micelles, and Targeted CCMs

#### 3.1.1. Characterization of Homopolymers and CCMs

**Figure 1:**
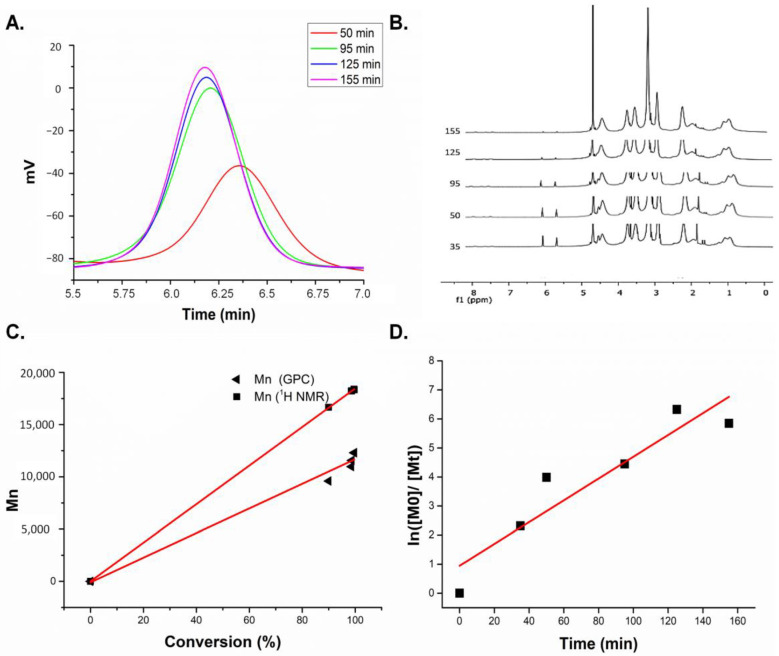
Evolution of GPC chromatograms of sulfobetaine at different times of RAFT polymerization acquired from LS detector (concentrations of all samples were identical in GPC analyses). (**A**) ^1^H NMR spectrum of sulfobetaine at different times. (**B**) Relationship between molecular weight and monomer conversion of sulfobetaine polymerizations. (**C**) Kinetics of RAFT polymerization of sulfobetaine (**D**).

In the first step, sulfobetaine homopolymer, as a macroCTA, was synthesized by RAFT polymerization. As explained in the Section 2.2, molecular weight and conversion analyses were performed to show whether the reaction was carried out by the RAFT mechanism. The molecular weight and conversion analyses were carried out by GPC and ^1^H-NMR spectroscopy, respectively. Figure 1A shows the GPC chromatograms of the homopolymers’ molecular weights, analyzed at certain time intervals by taking samples from the reaction medium. A clear shift in the molecular weight of the samples is shown by GPC, as the overlapping molecular weight distributions are shown [49]. Also, Figure 1B shows the ^1^H-NMR spectrum of the homopolymers taken from the reaction medium while the reaction is in progress. A decrease in monomer peaks (vinyl protons) shown at 5.8 and 6.2 was seen as the polymerization time increased. Figure 1C, D shows the conversion-time and molecular weight-conversion relationships drawn with the obtained data, and the relative amount of monomer/CTA/initiator, that increased linearly, was determined as 65/1/0.2.

**Figure 2:**
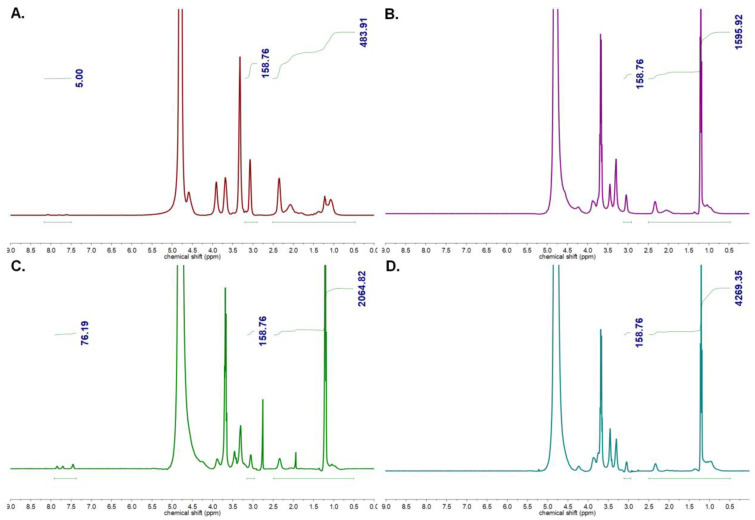
^1^H NMR spectrum of macroCTA (**A**), CCMs (**B**), peptide conjugated CCMs (**C**), and Herceptin conjugated CCMs (**D**) in D_2_O.

Following this step, we characterized the polymers and micelles by ^1^H-NMR. Although homopolymer and CCM structural characterizations by ^1^H-NMR were performed in our previous study [37], we used these data for comparison and to understand the efficiency of peptide- and antibody-binding to the CCMs. Due to this reason, we obtained ^1^H-NMR spectra of the homopolymer and CCM again and have reported it here. As discussed thoroughly in our previous study, Figure 2A shows the homopolymer’s (macroCTA’s) ^1^H NMR spectrum, in which the polymer backbone and CH_2_CH_2_SO_3_−, of SBMA’s side chain, signals are seen at 0.8–2.5 ppm. SBMA’s side chain’s signals are also seen at 2.96–3.13, 3.22–3.38, 3.80–4.00, and 4.46–4.65 ppm, respectively. The RAFT-ended group signal is seen at 7.49–8.20 ppm. For the CCMs’ ^1^H NMR spectrum, which is shown in Figure 2B, signals of the polymer backbone and -CH_2_CH_2_SO_3_− of SBMA’s side chain were observed at 0.8–2.5 ppm [50]. The signal of CTA end groups was not seen in the ^1^H NMR spectrum of CCMs, because of the higher molecular weight of CCMs, and the RAFT end group remaining in the internal structure of the micelle, as also discussed in our previous study [37]. As mentioned above, ^1^H NMR analysis of the homopolymer and micelle were utilized for the characterization of targeted NCs (as given in Figure 2A,B).

#### 3.1.2. Characterization of Peptide and Antibody Conjugated CCMs

In this study, an HER2-specific peptide and antibody (Herceptin) were conjugated to the CCMs, to obtain targeted nanocarriers, with the aim of comparing the peptide and antibody targeting. In order to do that, the LTVSPWY peptide was attached to the CCMs structure, due to its reported HER2-binding ability with weak immunogenic properties, and Herceptin was attached to the CCMs to obtain an antibody-based targeting molecule. Conjugation of the hydrophobic peptide to the nanoparticles might cause solubility problems due to its amino acid content. PEG-coupling to the LTVSPWY peptide has been applied to overcome this problem, resulting in better aqueous solubility characteristics [36,51,52]. In our case, instead of using PEG-coupling to LTVSPWY, we directly conjugated LTVSPWY peptides towards the carboxylic group into the polysufobetaine shell part of the micelles. However, this drove us to use varying amounts of peptides to find the peptide conjugated micelle with optimum size and solubility properties.

It is also well known that the conjugation of HER2 antibodies causes an increase in nanocarrier size. Additionally, due to the high molecular weight of Herceptin, the nanoparticles may cause precipitation, due to the increased nanoparticle molecular weight upon Herceptin binding. Therefore, we decided to use different amounts of antibodies, to find antibody conjugated micelles with good properties in terms of size and solubility. According to the literature, various amounts of Herceptin have been used to obtain Herceptin conjugated nanoparticles. Peng et al. used a molar ratio of the aldehyde group of polymers to amino groups of 5:1, due to one Herceptin molecule containing 66 free primary amino groups; Fiandra et al. incubated nanoparticles (1 mg) at room temperature for 2 h in the presence of Herceptin (0.3 mg) [20,53].

Here, we obtained a series of peptide- (PC1-PC5) and antibody-conjugated (AC1–AC5) CCMs by changing their mass ratios, as seen in Table 1. ^1^H-NMR, FTIR, and fluorescence spectroscopies were used for the characterization of these targeted micelles, and the results are given in Table 1. ^1^H-NMR spectra of a series of peptide- (PC1–PC5) and antibody-conjugated (AC1–AC5) CCMs are given in Supplementary Figures S1 and S2.

In the peptide conjugated CCMs’ (PC2) ^1^H NMR spectrum, shown in Figure 2C, besides the peaks belonging to the structure of the CCMs, new peaks were obtained at 1.46, 7.46, 7.71, and 7.85 ppm. While the signals of CTA end groups were not seen in the ^1^H NMR spectrum of CCMs, due to its high molecular weight, these aromatic signals were seen at 7.46, 7.71, and 7.85 ppm in the spectrum of peptide-bound CCMs originating from the tryptophan, tyrosine, and proline amino acids of the peptide. Similarly, Jie et al. synthesized LTVSPWY peptide-modified magnetic nanoparticles and showed benzene protons in the tryptophan and tyrosine units at 8.32 ppm [36]. In the ^1^H NMR spectrum of antibody-conjugated CCMs shown in Figure 2D, amine-related peaks were not observed clearly. However, we noticed that signals of -CH_2_CH_2_SO_3_− of SBMA side chain were overlayed at 2.96–3.13 ppm in the ^1^H NMR spectra of macroCTA, CCMs, peptide conjugated CCMs, and antibody-conjugated CCMs, which are shown in Figure 2A–D. When the integration of -CH_2_CH_2_SO_3_− of SBMA side chain is calculated using RAFT end group signal set to be 5, which is shown at 7.49–8.20 ppm from macroCTA’s ^1^H NMR spectrum, the integration peak corresponding to the SBMA signal was calculated to be 158.76 (equivalent to 2H from the group). Then we set the 2.96–3.13 signal as 158.76 for CCMs, antibody-conjugated CCMs, and peptide-conjugated CCMs, the integration of polymer backbone peaks, which was shown at 0.5–2.5 ppm in CCMs, peptide conjugated CCMs, and antibody conjugated CCMs (AC3) were 1595.92, 2064.82, 4269.35, respectively. This shows that this increase in integration in the polymer backbone is due to the binding of the peptide and antibody to the carboxyl group at the end group of the micelle, which also confirmed the conjugation of peptide and antibody to the micelles. All AC1–AC5 and PC1–PC5 series’ ^1^H NMR spectra are given in Figure S1 and Figure S2, respectively.

**Figure 3:**
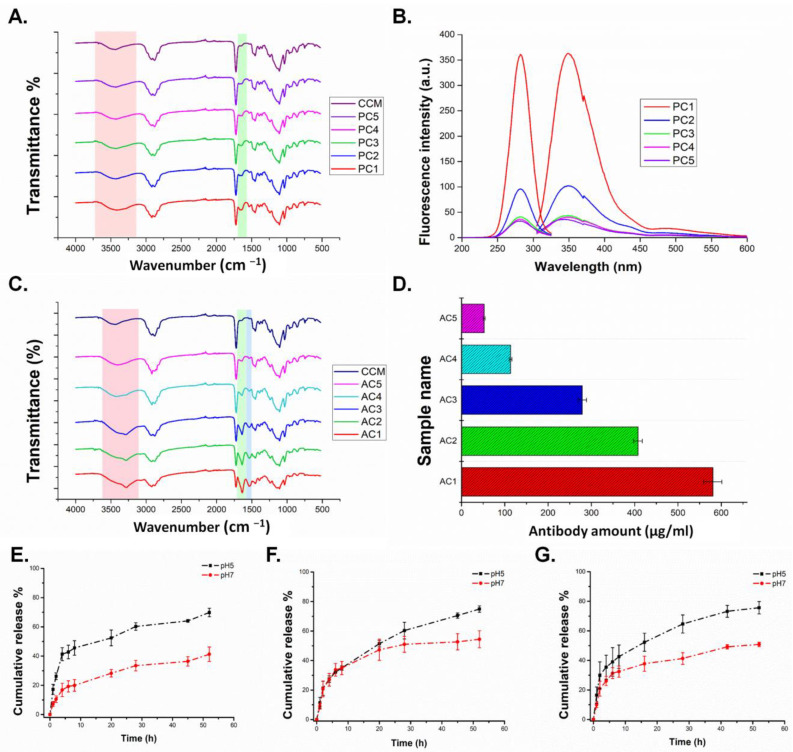
(**A**) FTIR spectra of PC1-5 and CCMS. (**B**) Excitation and emission spectrum of peptide conjugated PCs. (**C**) FTIR spectrum of AC1-5 and CCMS. (**D**) Antibody amount of ACs. (**E**) Release graph of micelles. (**F**) Peptide conjugated micelles. (**G**) Antibody conjugated micelles.

After confirming the structure with ^1^H NMR, we continued with FTIR spectroscopy. In the FTIR spectra of CCMs, peptide, and antibody-conjugated CCMs, which are shown in Figure 3A,C, the peak intensities at 3450 cm^−1^, assigned to O–H bending, and at 1600 cm^−1^, assigned to N–H bending, increased due to the addition of peptide and Herceptin to the micelles compared to the CCMs. Moreover, the intensity of the peaks increased as the proportion of peptide and Herceptin conjugated to the micelles increased. This might be explained by an increase in the N–H bonds arising from the peptide and antibody.

After confirming the structure with ^1^H NMR and FTIR spectroscopies, we proceeded with fluorescence spectroscopy, to determine the peptide and antibody amounts of the PC and AC series. Since our peptide sequence has amino acids (tryptophan and tyrosine) as a fluorescent feature, fluorescence scanning of the peptide (LTVSPWY) was performed to find the excitation and emission values with a 1 mg/mL concentration of peptide solution in DMSO. Here, the value where the peptide gave the highest absorbance was determined as 280 nm, which has also been shown to be the absorption value of tryptophan and tyrosine amino acids [54]. Then, we used this value as an excitation value of the peptide and performed emission scanning, following this step, based on the highest emission value obtained from this scanning. Thus, the necessary Ex/Em values to measure the concentration of our peptide-bound micelle were determined as 280/350 nm. Afterward, 1–100 µg/mL concentration of the peptide solutions in DMSO were prepared, and a calibration graph was obtained (Supplementary Figure S3). PC1–PC5 and CCMs, as control group solutions, were prepared, and the peptide amount of these micelles was determined using this calibration graph. According to the fluorescence screening, which is shown in Figure 3B, the PC1 sample with the highest peptide ratio gave the highest fluorescence values, while a decrease in fluorescence values occurred when going towards PC5, and the fluorescence values of the PC4 and PC5 samples were close to the values of the CCMs. After this scanning process, the peptide amounts of the peptide-bound micelles were calculated as µg/mL using the fluorescence values obtained at Ex/Em 280/350 nm, and are given in Table 1. In order to determine the Herceptin amount in the micelles, a BCA assay was performed. Figure 3D shows the amount of AC1–5 and CCMs with a 1 mg/mL concentration. According to these results, the AC1 sample had the highest amount of Herceptin, with 600 µg/mL, which is approximately 50% (*w*/*w*) of the micelles, and the amount of Herceptin decreased in the AC5 sample.

Following this chemical characterization, we determined the size and charge of the antibody and peptide micelles. Table 1 shows the size and zeta potential of the AC1–5 samples. The AC1–2 samples had the largest size at 400 nm, and 300 nm, respectively, and their PDI (polydispersity index) values are very high. Since the increase in the Herceptin ratio caused the molecular weights of the AC1 and AC2 samples to increase, a tendency to precipitate was observed in the solutions of these samples (Supplementary Figure S4). However, it was observed that the AC3–5 samples exhibited superior characteristics in terms of size and solubility properties when compared to the AC1 and AC2 samples. Zhao et al. and Bolu et al. showed that Herceptin increased micelles’ size and PDI values with conjugation [55,56]. Furthermore, it was observed that the zeta potential of Herceptin-conjugated micelles decreased as the ratio of Herceptin conjugation increased. Peng et al. also showed this type of phenomenon in their study. It might be explained by the increased number of –COOH groups on the micelles’ surfaces, which reduces the micelles’ charges [20]. Since this study aimed to show the importance of targeting, we conducted our study with the AC3 samples, with high antibody content, good solubility, size, and charge properties (90 ± 40 nm, 10.2 ± 4.3 mV, 279 ± 9.5 µg/mL antibody).

For peptide-conjugated micelles, the PC1 samples had a higher size, and the micelle size decreased as the amount of peptide decreased (Figure S5). However, the higher size of PC1, and higher hydrophobic peptide content of these micelles, caused a solubility problem. Also, the zeta potential of peptide-conjugated micelles decreased with an increased peptide ratio. It also has the same trend with antibody-conjugated micelles. Compared with PC1, the PC2–5 samples had a good size, PDI, and solubility features. According to these data, we decided to continue with PC2, the most suitable sample, with high peptide content, good solubility, size, and charge properties. In summary, peptide and antibody conjugation increased micelle size, as CCMs have the size of 65.5 ± 6.2 nm, which is shown in Supplementary Figures S4 and S5. Besides, SEM images of AC1, AC3, and PC2 are shown in Supplementary Figure S6. CCMs’ sizes, determined by SEM and DLS spectrometry, have concurred with each other.

After the selection of AC3 and PC2 as the targeted CCMs, we proceeded to the drug-loading study. In this study, we used dialysis as a standard loading method. Based on this method, the drug amount can be calculated as either supernatant or pellet, to calculate drug loading and entrapment efficiencies. Once we calculated both methods, we noticed that the supernatant method’s loading and entrapment efficiencies were higher than the pellet ones. The difference between the two methods could be due to the adhesion of free DOX to the dialysis membrane, affecting its penetration to the supernatant. Therefore, we decided to proceed by pellet, which is more accurate than the supernatant procedure, and calculated the loading and entrapment efficiency with this method. The drug loading efficiencies of CCMs, PC2, and AC3 were 26, 50, and 56%, respectively, and the entrapment efficiencies of CCMs, PC2, and AC3 were 1.7, 2, and 3.1, respectively. Conjugation of the peptide and antibody increased their drug loading efficiency and entrapment efficiency. This might be explained by the hydrophobic peptides interacting with more of the hydrophobic doxorubicin, and the higher molecular weight of antibodies providing more interaction sites for doxorubicin, resulting in an increase in the loading and entrapment efficiencies.

#### 3.1.3. Release Study

Since we designed acid-sensitive core cross-linked micelles, we performed a release study in acidic and neutral environments (Figure 3E–G). According to the release graphs of the peptide and antibody-linked formulations, a varying release regime was observed at acidic and neutral pH. Peptide/antibody conjugation caused some changes in the release profiles. This proves the contribution of polymeric components of the non-targeted nanocarrier’s release behavior. In the peptide-conjugated CCMs, a delayed drug release was obtained. In all three cases, drug release in acidic media is associated with cleavage of acetal bonds and with the zwitterionic character of sulfobetaine and its interaction with both DOX and the environment due to this feature. It can be expected that sulfobetaine, since it is negatively charged, has a lower preference for interacting with an acidic medium at pH 4.5, interacting with DOX (pKa > 7) and delaying the release in an acidic medium. In addition, it is a possibility that negatively charged sulfobetaine chains prefer to interact with the medium at neutral pH, and the release increases at this pH. It was seen that the release of DOX molecules is slightly higher at pH 7 with the addition of additional molecules, such as peptides or antibodies, to the structure. This may be because DOX also interacts with the peptide or antibody on the carrier during loading, which was also released during release. In addition, the presence of molecules such as peptides/antibodies on the nanocarrier may cause a slight delay in the release, by slowing down the solution entry into the structure, as reported in previous studies [57,58]. In summary, it can be said that, although a higher release is observed in the acidic medium due to peptide and antibody binding, the presence of peptides and antibodies causes lower than expected release. However, acid-induced release was observed in all three types of carriers.

### 3.2. Cell Studies

#### 3.2.1. Determination of Cytotoxic Effects of CCMs on Cells by MTT Assay

**Figure 4:**
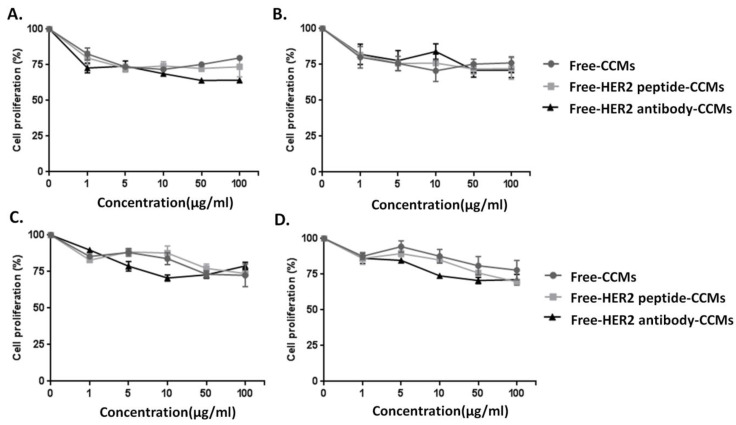
Cytotoxic effect of drug-free CCMs, HER2-peptide and HER2-antibody CCMs (0–100 µg/mL) on 48 and 72 h SKBR-3 breast cancer (**A**: 48 h; **B**: 72 h) and MCF-10A (**C**: 48 h; **D**: 72 h) cells.

The cytotoxic effects of drug-free CCMs (without drug), and their targeted forms, (HER2-CCMs) on SKBR-3 and MCF-10A at 48 and 72 h were determined by MTT assay. Based on Figure 4, the proliferation of the cells was around 75-100% at concentrations from 0.1 to 100 µg/mL of CCMs, at both 48 and 72 h of incubation. Also, the cell proliferation of SKBR-3 and MCF-10A cells was slightly dependent on the amount of CCMs.

Furthermore, the cytotoxicity effect of DOX-loaded CCMs (DOX-CCMs, DOX-HER2 peptide-CCMs, and DOX-HER2 antibody-CCMs) was evaluated on SKBR-3 and MCF-10A cells. Based on our previous study, the cytotoxic effect of DOX was determined to find the optimal concentration of DOX-loaded CCMs. Incremental increases in the concentration of DOX (0.1–5 μM) for SKBR-3 cells, and DOX (0.05–1 μM) for MCF-10A cells, were performed to determine the inhibitory concentration (IC50) value at 48 h. The IC50 values of DOX at 48 h were 0.65 μM and 0.08 µM for SKBR-3 and MCF-10A cells, respectively [19]. These concentration ranges of DOX-CCMs, DOX-HER2 peptide-CCMs, and DOX-HER2 antibody-CCMs were used to determine the cytotoxic effects on the SKBR-3 and MCF-10-A cell lines at 48 and 72 h, by MTT cell proliferation test.

**Figure 5:**
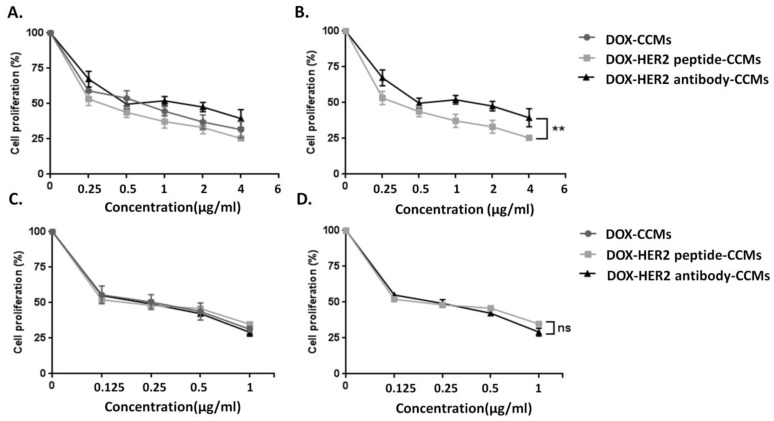
Cytotoxic effect of DOX-CCMs, DOX-HER2 peptide-CCMs, and DOX-HER2 antibody-CCMs (0–4 µM) on SKBR-3 (**A**,**B**) and (0–1 µM) MCF-10A (**C**,**D**) cells for 48 h. Note that, in all cytotoxicity analyses, experiments were performed in triplicate and repeated three times under similar conditions, and paired *t*-test was performed for statistical analysis, and *p* < 0.01:** were considered significant. Non-significant was shown as ns.

The IC50 values of DOX-CCMs, DOX-HER2 peptide-CCMs, and DOX-HER2 antibody-CCMs were 0.71, 0.34, and 0.49 µM, respectively, for SKBR-3 cells at 48 h (Figure 5A,B and Table 2). A remarkable change and differences were demonstrated for both DOX-HER2 peptide-CCMs and DOX-HER2 antibody-CCMs (Figure 5B and Table 2). Comparing the IC50 values, DOX-HER2 peptide-CCMs were 2-times more effective on SKBR-3 cells as compared to DOX-HER2 antibody-CCMs, which indicates that the targeting efficiency of the HER2-specific peptide is greater than the DOX-HER2 antibody-CCMs (Figure 5A,B and Table 2). Also, conjugation of peptides and antibodies to CCMs did not lead to any difference in selectivity for MCF-10A cells, which demonstrates that there was no preferential uptake of targeted CCMs by healthy cells, according to the IC50 values (0.27, 0.23, and 0.19 µM for MCF-10A cells of DOX-CCMs, DOX-HER2 antibody-CCMs, and DOX-HER2 peptide-CCMs at 48 h, respectively) (Figure 5C,D and Table 2). This means that DOX-HER2 antibody-CCMs and DOX-HER2 peptide-CCMs exerted similar cytotoxic effects on MCF-10A. The effects of DOX-HER2-peptide and DOX-HER2-antibody CCMs were higher on HER2 overexpressed SKBR-3 cells compared to DOX-CCMs.

**Figure 6:**
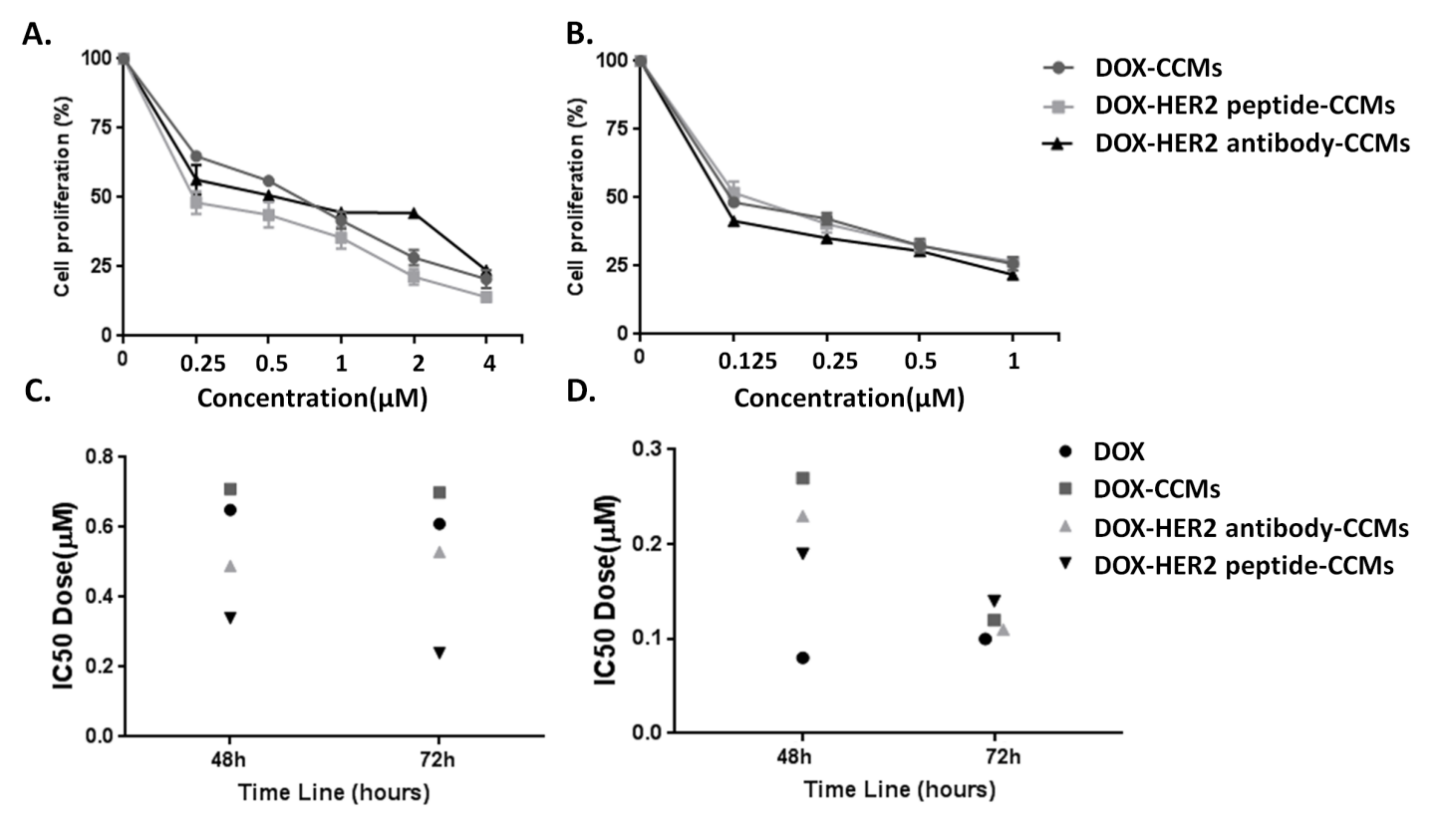
DOX-CCMs, DOX-HER2 peptide-CCMs, and DOX-HER2 antibody-CCMs (0–4 µM) on 72 h SKBR-3 breast cancer (**A**) and (0–1 µM) cytotoxic effect on MCF-10A (**B**) cells and SKBR-3 (**C**) and MCF-10A (**D**) IC50 dose distribution of cells at 48 and 72 h.

Peng J. et al. and Kumar A. et al. reported that DOX-loaded HER2 antibody (Trastuzumab or Herceptin)-conjugated micelles had a potential or promising role on HER2-positive breast cancer cells, in terms of increasing the efficiency of targeted nanoparticles [20,59]. In our studies we have aimed at increasing targeting, and delivering potent cancer drug carrying micelles, by using peptides and antibodies to affect cancer cells, thus minimizing toxic side effects. For this purpose, DOX-HER2 antibody-CCMs and DOX-HER2 peptide-CCMs were applied and compared in terms of cytotoxicity. Also, the IC50 value of 0.34 µM for DOX-HER2 peptide-CCMs on SKBR-3 cells, showed that DOX-loaded HER2 CCMs enhanced the effect of DOX compared to both DOX alone and DOX-CCMs, at 48 h. Besides that, we observed that targeted micelles have less toxicity on MCF-10A cells, according to the IC50 values of DOX-loaded CCMs. This suggests that using CCMs with/without peptides and antibodies elicits a kind of masking effect on toxicity by DOX. This is a significant result, proving the increased biocompatibility caused by nanocarriers. Similarly, Sheng et al. observed lower toxicity in healthy cells than in naked DOX molecules. They also reported that MCF10-A is more susceptible to all formulations, supporting our findings [60]. Also, the results showed that the IC50 values of DOX-CCMs, DOX-HER2 peptide-CCMs, and DOX-HER2 antibody-CCMs were 0.7, 0.24, and 0.53 µM for SKBR-3 cells at 72 h (Figure 6A and Table 2), and 0.12, 0.14, and 0.11 µM for MCF-10A cells at 72 h (Figure 6B and Table 2), respectively. According to the IC50 values, DOX-HER2 peptide-CCMs were 2-times more effective on SKBR-3 cells as compared to DOX-HER2 antibody-CCMs, as well as around 3-times more effective on SKBR-3 cells as compared to DOX-HER2 peptide-CCMs. However, using peptides or antibodies with CCMs did not affect the MCF-10A cells in terms of selectivity. At the same time, the distribution of IC50 values for SKBR-3 (Figure 6C and Table 2) and MCF-10A (Figure 6D and Table 2) at 48 h and 72 h showed that DOX-HER2 peptide-CCMs, at both 48 h and 72 h, were more effective on SKBR-3 cells compared to DOX-HER2 antibody-CCMs. However, using peptides or antibodies with CCMs has been shown not to have selectivity potential for MCF-10A cells.

#### 3.2.2. Determination of Uptake Amount of CCMs by Cells with Fluorescence Imaging

**Figure 7:**
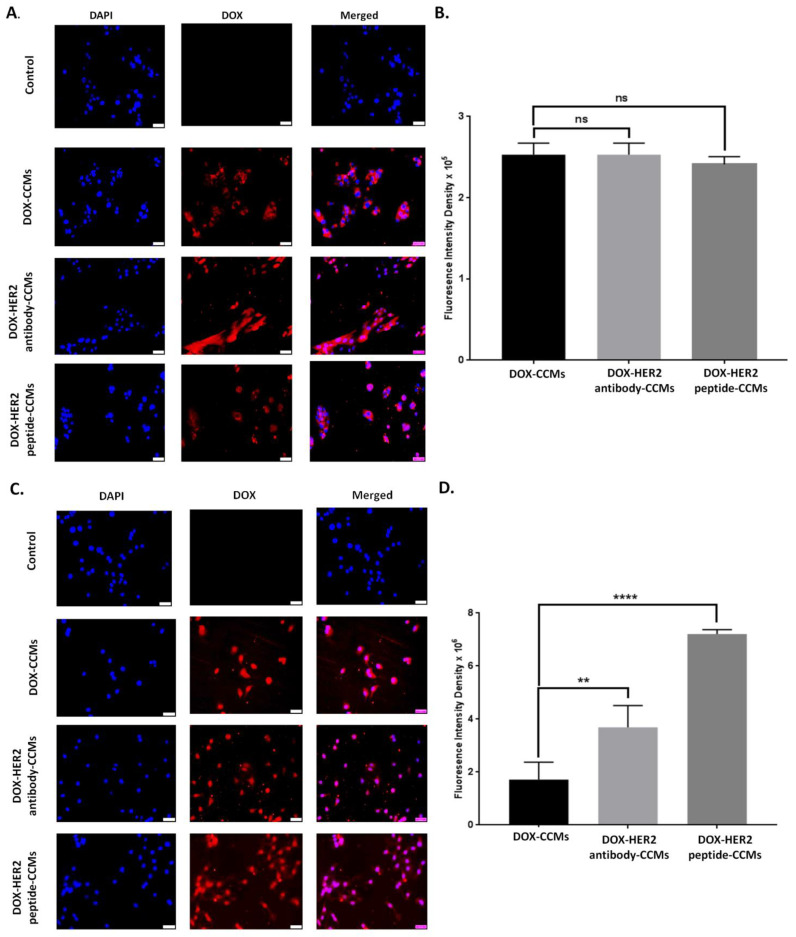
Fluorescence images and fluorescence intensity density of DOX-loaded HER2-peptide CCMs and HER2-antibody CCMs on MCF-10A (**A**,**B**) and SKBR-3 (**C**,**D**) cells at 48 h, Magnification: 20×, Blue: DAPI; Red: DOX. All experiments were tested and analyzed in triplicate, and repeated three times with similar conditions and paired *t*-test was performed for statistical analysis, and *p* < 0.01:**; *p* < 0.0001:**** were considered significant for 10 different image sections. The error bars represent the standard deviations. Non-significant was shown as ns.

The uptake rates of 0.34 µM of DOX-CCMs, DOX-HER2 peptide-CCMs, and DOX-HER2 antibody-CCMs by SKBR-3 cells at 48 h were analyzed by fluorescence imaging (Figure 7A–D). For this, ten different images were analyzed using the ImageJ software. The concentration of the IC50 values of DOX-HER2 peptide-CCMs for SKBR-3 cells was applied to determine the uptake amount of CCMs. It was shown that the fluorescence intensity density values of the DOX-HER2 peptide-CCMs and DOX-HER2 antibody-CCMs were higher as compared to DOX-CCMs. Also, compared to the DOX-HER2-peptide-CCMs and DOX-HER2-antibody-CCMs, DOX-HER2 peptide-CCMs have higher uptake rates (Figure 7D).

After unpaired statistical analysis, the fluorescence intensity density values of DOX-HER2 peptide-CCMs and DOX-HER2 antibody-CCMs were four-fold and two-fold greater than DOX-CCMs on SKBR-3 cells, respectively. This difference was also significant. MCF-10A cells were treated with the IC50 value of CCMs, and the results showed that the fluorescence intensity density value of DOX-CCMs, DOX-HER2 peptide-CCMs, and DOX-HER2 antibody-CCMs were similar (Figure 7B), due to the absence of the HER2 receptor on MCF-10A cells. This result revealed that the DOX-HER2 peptide-CCMs were more effective than DOX-CCMs and DOX-HER2 antibody-CCMs on SKBR-3 cells, which can be clarified by greater intracellular uptake of DOX molecules.

#### 3.2.3. Determination of Apoptotic Effects of CCMs on Cells

The IC50 values of DOX-CCMs, DOX-HER2-peptide-CCMs, and DOX-HER2 antibody-CCMs were applied to SKBR-3 cells for 48 h to determine apoptosis and necrosis percentages by Annexin V/PI double staining assay. Moreover, the expression levels of pro-apoptotic and anti-apoptotic proteins were determined with Western blotting.

**Figure 8:**
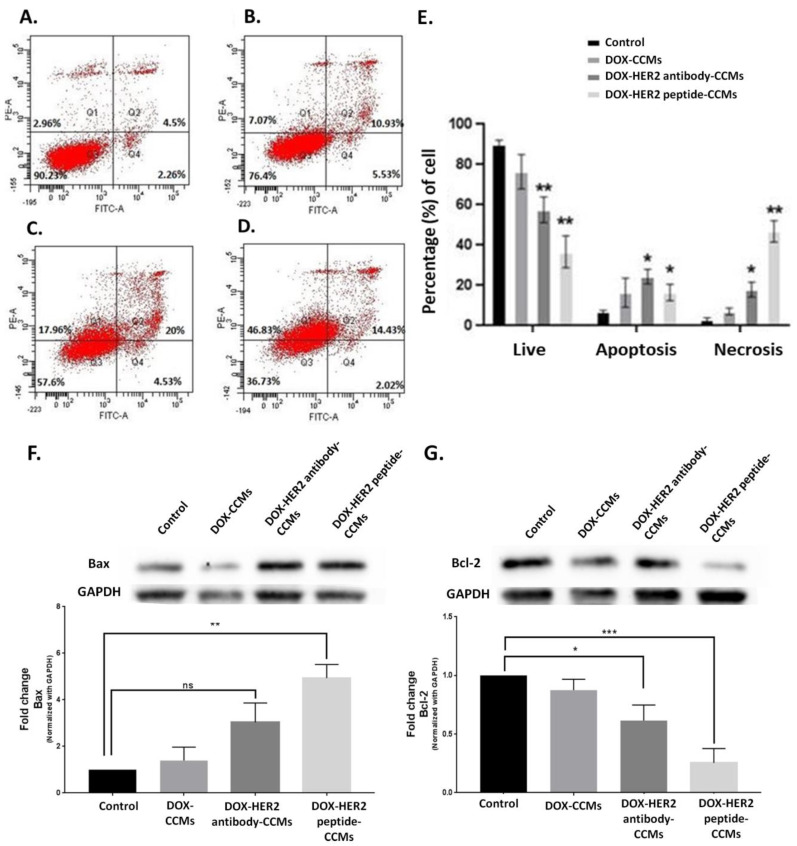
The apoptotic effects of the control (**A**), IC50-loaded DOX molecule-loaded (**B**), DOX-loaded HER2-peptide CCMs (**C**), HER2-antibody CCMs (**D**) with Annexin V/PI double staining and protein expression level of Bax (**F**) and Bcl-2 (**G**) with Western blotting on the SKBR-3 breast cancer cells after 48 h of incubation. (**E**) Percentage (%) of live, apoptotic, and necrotic cells after treatment with DOX-CCMs, DOX-HER2 antibody CCMs, and DOX-HER2 peptide CCMs. Note that, these experiments were performed in triplicate and repeated three times under similar conditions, and paired *t*-test was performed for statistical analysis, and *p* < 0.05:*, *p* < 0.01:**, and *p* < 0.001:*** were considered significant. Non-significant was shown as ns.

A 0.34 µM dose (IC50 dose of DOX-HER2 peptide-CCMs) of DOX-CCMs, DOX-HER2 peptide-CCMs, and DOX-HER2 antibody-CCMs increased the percent of the apoptotic cell population, as well as induced necrosis (Figure 8A–F). Low doses of DOX-HER2 peptide-CCMs were more effective than DOX-CCMs and DOX-HER2 antibody-CCMs on SKBR-3 cells. Bcl-2 and Bax proteins have a role in the regulation of apoptotic cell death pathways. For this reason, in order to determine the anti-apoptotic and pro-apoptotic protein levels, Bcl-2 and Bax proteins were used [61,62]. Investigating Bax and Bcl-2 in Western blot experiments is often used as a way to assess the balance between pro- and anti-apoptotic signals in cells. In this experiment, GAPDH was used as an internal loading control to understand the changes in protein levels [63].

Doxorubicin, which we used as a chemotherapeutic agent in this study, induces apoptosis in tumor cells. In this step, 0.34 µM (IC50 value) of DOX-CCMs, DOX-HER2 peptide-CCMs, and DOX-HER2 antibody-CCMs were applied on cells. Since we used CCMs, CCMs-peptide, and CCMs-antibody to target the cells, and showed peptide- and antibody-conjugated CCMs provided a greater uptake of these CCMs on the cancer cells, we wanted to determine and confirm whether the DOX uptake change or not the apoptosis-related proteins. The results demonstrated that there was a 3-fold decrease in the levels of Bcl-2 in response to DOX-HER2 peptide-CCMs treatment, as well as a 1-fold decrease for DOX-HER2 antibody-CCMs treatment (Figure 8G). Also, the Bax protein level, after the application of DOX-HER2 antibody-CCMs, increased, but it was not significant. However, the protein level of Bax increased 2-fold in response to treatment with DOX-HER2 peptide-CCMs (Figure 8F). These results were supported by a mitochondrial membrane potential assay (JC-1 assay) in Supplementary Figure S7.

In the literature, there are several studies which show that targeted nanoparticles robustly inhibit and decrease cell proliferation by triggering apoptosis [64,65]. In our studies, it was found that targeted nanoparticles induced an apoptotic pathway as a result of activation of the apoptotic protein, Bax, or a reduction in the anti-apoptotic protein, Bcl-2 (Figure 8F,G).

**Figure 9:**
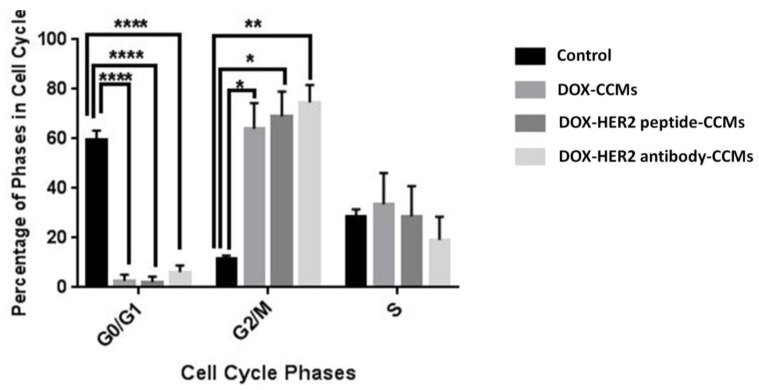
Determination of cytostatic effects of the IC50-loaded DOX-loaded CCMs, DOX-loaded HER2-peptide CCMs, and DOX-loaded HER2-antibody CCMs on the SKBR-3 breast cancer cells after 48 h of incubation. Note that, these experiments were performed in triplicate and repeated three times under similar conditions, and paired *t*-test was performed for statistical analysis, and *p* < 0.05:*, *p* < 0.01:**, and *p* < 0.0001:**** were considered significant.

It is well-known that DOX has cytotoxic effects as an inhibitor of topoisomerases II, resulting in G1 and G2 cell cycle arrest and increased apoptosis [66]. It was hypothesized that the DOX-loaded CCMs would affect the cell cycle. SKBR-3 cells treated with 0.34 µM (IC50 dose of DOX-HER2 peptide-CCMs) of DOX-CCMs, DOX-HER2 peptide-CCMs, and DOX-HER2 antibody-CCMs increased the G2/M phase and decreased the G0/G1 phase of the cell cycle, that also show significant changes (Figure 9). The important point is that DOX-HER2 peptide-CCMs were more specific and selective for SKBR-3 cells than DOX-CCMs and DOX-HER2 antibody-CCMs in our study.

After cytotoxic, apoptotic and cytostatic assays, the genotoxic effect of DOX-CCMs, DOX-HER2 peptide-CCMs, and DOX-HER2 antibody-CCMs were demonstrated by comet assay [67].

**Figure 10:**
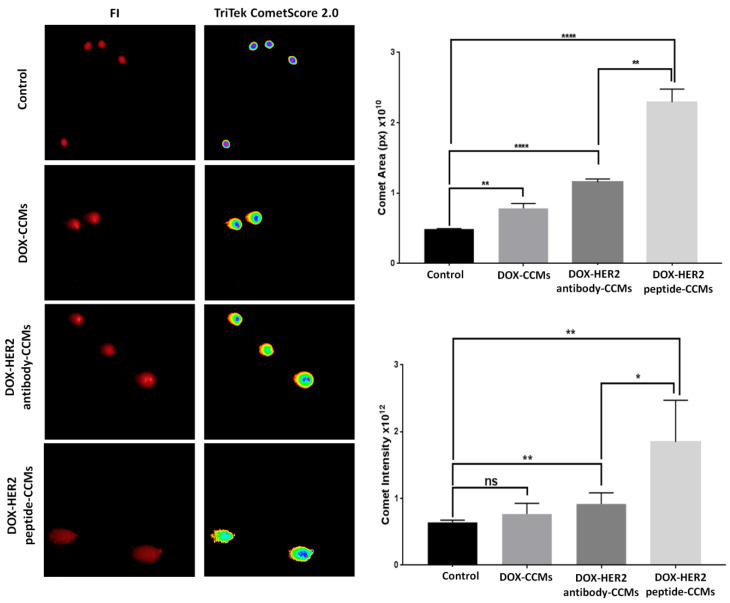
Analysis of the genotoxicity of the IC50-loaded DOX-molecule-loaded, DOX-molecule-loaded HER2 targeting peptide (LTVSPWY) and monoclonal antibody (Herceptin^®^) on SKBR-3 breast cancer cells after 48 h incubation. (FI: Propidium iodide (red) TriTek CometScore 2.0: Fluorescence dye density (multi-to-pink; pink–blue–green–yellow–red)). CometScore is freely available software tool developed bz Rex Hoover (Sumerduck, VA, USA). Note that, these experiments were performed in triplicate and repeated three times under similar conditions, and paired *t*-test was performed for statistical analysis, and *p* < 0.05:*, *p* < 0.01:**, and *p* < 0.0001:**** were considered significant. Non-significant was shown as ns.

SKBR-3 cells treated with 0.34 µM (IC50 dose of DOX-HER2 peptide-CCMs) of DOX-CCMs, DOX-HER2 peptide-CCMs, and DOX-HER2 antibody-CCMs induced DNA fragmentation that increased comet formation (Figure 10). A treatment of 0.34 µM of DOX-HER2 peptide-CCMs applied to SKBR-3 cells led to a higher comet area and comet intensity compared to DOX-CCMs and DOX-HER2 antibody-CCMs.

## 4. Discussion

In this study, we prepared a polysulfobetaine-based, breast-cancer-targeted CCMs, with pH sensitivity. All the physicochemical analyses showed that uniform CCMs were obtained by RAFT polymerization, with 65.5 ± 6.2 nm size and 14.5 ± 0.7 mV charge, which enables controlled and one-step synthesis of the particles. Following this step, we conjugated these CCMs with different amounts of the LTVSPWY peptide or Herceptin, to obtain targeted CCMs, and characterized their physicochemical properties by ^1^HNMR, FTIR, fluorescence spectrophotometer, and Zetasizer. The conjugation of antibody or peptide increased the size of the CCMs, causing solubility problems. Due to this, we synthesized different concentrations of the LTVSPWY peptide (13.84, 1.38, 0.55, 0.27, and 0.14 mg) and Herceptin (60, 30, 15, 5, and 1 mg) containing-CCMs to obtain optimum formulations in terms of size, charge, solubility, and higher ligand contents. We obtained formulations with sizes ranging between 235 ± 127 and 113 ± 17 nm, and zeta potentials of 9.15 ± 3.3–14.7 ± 3.6 for peptide-conjugated CCMs, and with sizes ranging between 428 ± 113 and 78 ± 38, and zeta potentials of 10.2 ± 4.3–16.2 ± 3.8 mV for antibody-conjugated CCMs. Although a higher amount of peptides and antibodies is desired for the targeted nanoparticles, in our case, solubility problems were caused when we increased the amount of antibody and peptide, as the antibody and peptide were conjugated with the hydrophilic -COOH group of the sulfobetaine. For this reason, we proceeded to PC2 and AC3 formulations, which were 141 ± 60 and 90 ± 40 nm in size, and 13.4 ± 3.8 and 10.2 ± 4.3 mV in charge, respectively, for further studies. Following this step, we performed a release study at pH 7.4 and pH 5, and showed that CCMs, peptide-, and antibody-conjugated CCMs are stable at physiological pH, and provide acid-induced drug release with an acetal-based cross-link of the micelle core. Although the presence of molecules such as peptides/antibodies on the CCMs caused a slight delay in release, we achieved approximately 80% release at acidic pH within the first 48 h, providing targeted release to cancer tissue by the acid effect. Following physicochemical characterization, we proceeded with the in vitro cell experiment, and investigated and compared the efficacy of the HER2 peptide (LTVSPWY) and HER2 antibody (Herceptin^®^) conjugated micelles by cytotoxic, apoptotic, cytostatic, and genotoxic assays. The cytotoxicity of the targeted CCMs was determined on SKBR3 cells with a control of MCF-10A. The IC50 values of DOX-CCMs, DOX-HER2 peptide-CCMs, and DOX-HER2 antibody-CCMs were 0.71 μM, 0.34 μM, and 0.49 μM, respectively, for SKBR-3 cells after 48 h. The DOX-HER2 peptide-CCMs were 2-times more effective on SKBR-3 cells compared to DOX-HER2 antibody-CCMs. The IC50 values for MCF-10A cells were 0.27 μM, 0.23 μM, and 0.19 μM for DOX-CCMs, DOX-HER2 antibody-CCMs, and DOX-HER2 peptide-CCMs, respectively, showing no difference in selectivity for healthy cells. The effects of DOX-HER2-peptide and DOX-HER2-antibody conjugated CCMs were higher on HER2 overexpressed SKBR-3 cells compared to DOX-CCMs. The fluorescence intensity density values of DOX-HER2 peptide-CCMs and DOX-HER2 antibody-CCMs were higher compared to DOX-CCMs for SKBR-3 cells. The fluorescence intensity density value of DOX-HER2 peptide-CCMs was four-fold more, and that of DOX-HER2 antibody-CCMs was two-fold more, compared to DOX-CCMs. However, for MCF-10A cells, the fluorescence intensity density values of DOX-CCMs, DOX-HER2 peptide-CCMs, and DOX-HER2 antibody-CCMs were similar, due to the absence of HER2 receptors. This suggests that DOX-HER2 peptide-CCMs were more effective than the other two on SKBR-3 cells. The study also revealed that the effects of the peptide- and antibody-conjugated CCMs were similar on MCF-10A cells. This may indicate that the effectiveness of the conjugated CCMs is specific to HER2-overexpressed cells, and the conjugated peptide may be more effective in inhibiting the dimerization of the HER2 receptor, leading to apoptosis of the cancer cells. This suggests that using HER2-specific peptide- and antibody-conjugated CCMs could lead to better anticancer effects than DOX-CCMs, in cancer cells that overexpress HER2. The peptide conjugation seemed to have more advantage in terms of targeting efficiency compared to the antibody conjugation, as indicated by a 2-fold higher IC50 value for DOX-HER2 peptide-CCMs compared to DOX-HER2 antibody-CCMs, on SKBR-3 cells. However, no preferential uptake of the targeted CCMs was observed in the healthy cells, MCF-10A, due to the absence of HER2 receptors, as indicated by similar IC50 values for all three types of CCMs. We also evaluated the apoptotic effect of DOX-CCMs, DOX-HER2 peptide-CCMs and DOX-HER2 antibody-CCMs by controlling the apoptotic proteins Bcl-2 and Bax, and there was a 3-fold decrease in protein levels of Bcl-2 in response to DOX-HER2 peptide-CCMs treatment as well as a 1-fold decrease for DOX-HER2 antibody-CCMs treatment. In addition, the Bax protein level, after the treatment with DOX-HER2 peptide-CCMs, was increased by 2-fold, and while it was increased after the treatment of DOX-HER2 antibody-CCMs, it was not significant. These show that treatment with HER2-specific peptide-conjugated CCMs induced apoptosis and primary necrosis by specific apoptotic proteins that are proven with specific Bax and Bcl-2 antibodies. We also evaluated the cell cycle arrest of DOX-CCMs, DOX-HER2 peptide-CCMs, and DOX-HER2 antibody-CCMs. The percentage of the cells in the G0/G1 phases after the DOX-HER2 peptide-CCMs treatment was higher than for the DOX-CCMs and DOX-HER2 antibody-CCMs. Lastly, we evaluated the genotoxic effects of DOX-CCMs, DOX-HER2 peptide-CCMs, and DOX-HER2 antibody-CCMs by comet assay. DOX-CCMs, DOX-HER2 peptide-CCMs, and DOX-HER2 antibody-CCMs induced DNA fragmentation that increased comet formation, and DOX-HER2 peptide-CCMs applied to SKBR-3 cells had a higher comet area and comet intensity compared to DOX-CCMs and DOX-HER2 antibody-CCMs, meaning that DOX-HER2 peptide-CCMs had higher apoptotic and cytostatic effects on SKBR-3 cells, compared to DOX-CCMs and DOX-HER2 antibody-CCMs.

Moreover, the results emphasize the selective targeting ability of the HER2-specific peptide- and antibody-conjugated CCMs, which has the potential to enhance the therapeutic efficacy of the drugs, while reducing the side effects. Furthermore, the study found that peptide targeting had more advantages over antibody targeting, as the fluorescence intensity density value of DOX-HER2 peptide-CCMs was higher as compared to DOX-HER2 antibody-CCMs. This phenomenon implies that peptide conjugation could be a better option for HER2-positive cancer cell targeting.

It should be noted that further studies, with a larger sample size and longer follow-up period, are required to validate these findings, and to establish the clinical relevance of these results. Nevertheless, these results provide valuable insights into the targeting mechanism of peptide- and antibody-conjugated CCMs, and the potential benefits of these conjugates for the treatment of HER2-positive cancer cells.

## 5. Conclusions

In this study, we showed that the HER2-targeted peptide and antibody-based acid-degradable polysulfobetaine-based core cross-linked micelles selectively affected HER2-bearing tumor cells. Furthermore, compared to peptide- and antibody-conjugated CCMs, peptide-conjugated CCMs had superior physicochemical and targeting efficiencies than the antibody-conjugated CCMs. The present findings carry significant implications, as they illustrate that the utilization of a small and structurally stable peptide ligand is superior to the use of an antibody-based ligand, which may exhibit a loss of activity and high molecular weight. The present body of in vitro research indicates that polysulfobetaine-based core cross-linked micelles (CCMs) possess great potential as a treatment modality for HER2-positive breast cancer, as an alternative to PEG-based CCMs.

## Figures and Tables

**Table 1:** Size, size distribution, zeta potential, peptide, and antibody amount of micelles.

	**Size (nm)**	**PDI**	**Zeta Potential (mV)**	**Peptide Amount (µg/mL)**
CCMs	65.5 ± 6.2	0.269	14.5 ± 0.7	-
PC1	235 ± 127	0.345	9.15 ± 3.3	15.47 ± 0.49
PC2	141 ± 60	0.318	13.4 ± 3.8	3.19 ± 0.37
PC3	141 ± 33	0.415	13.8 ± 3.8	0.66 ± 0.09
PC4	118 + 43	0.482	14.7 ± 3.6	0.60 ± 0.27
PC5	113 ± 17	0.486	14.5 ± 3.7	0.34 ± 0.04
	**Size (nm)**	**PDI**	**Zeta Potential (mV)**	**Antibody amount (µg/mL)**
CCMs	65.5 ± 6.2	0.269	14.5 ± 0.7	-
AC1	428 ± 113	0.466	13.3 ± 5.4	580.4 ± 21
AC2	316 ± 119	0.499	16.2 ± 3.8	407.6 ± 10.2
AC3	90 ± 40	0.344	10.2 ± 4.3	279 ± 9.5
AC4	79 ± 45	0.287	11.4 ± 4.1	113.7 ± 3.0
AC5	78 ± 38	0.280	15.0 ± 4.7	52.33 ± 2.7

**Table 2:** Summary of the IC50 values of DOX-CCMs, DOX-HER2 peptide-CCMs, and DOX-HER2 antibody-CCMs on SKBR-3 and MCF-10A for 48 and 72 h incubation time.

	IC50 Value for 48 h Incubation Time	IC50 value for 72 h Incubation Time
**SKBR-3 cells**		
DOX-CCMs	**0.71** µM	0.70 µM
DOX-HER2 antibody-CCMs	0.49 µM	0.53 µM
DOX-HER2 peptide-CCMs	0.34 µM	0.24 µM
**MCF-10A cells**		
DOX-CCMs	0.27 µM	0.12 µM
DOX-HER2 antibody-CCMs	0.23 µM	0.11 µM
DOX-HER2 peptide-CCMs	0.19 µM	0.14 µM

## Data Availability

The data that supports the findings of this study are available from the corresponding author upon reasonable request.

## Supplementary Materials

The following supporting information can be downloaded at: https://www.mdpi.com/article/10.3390/pharmaceutics15030733/s1, Figure S1: 1H NMR of AC1(A), AC2(B), AC3(C), AC4(D), AC5(E), and CCMs(F) in DMSO; Figure S2: 1H NMR of PC1(A), PC2(B), PC3(C), PC4(D), PC5(E), and CCMs(F) in DMSO; Figure S3: Calibration graph of LTVSPWY peptide in DMSO; Figure S4: Size distributions of AC1(A) AC2(B) AC3(C) AC4(D) AC5(E) CCMs(F); Figure S5: Size distributions of PC1(A) PC2(B) PC3(C) PC4(D) PC5(E) CCMs(F); Figure S6: SEM images of selected samples (AC1, AC3, and PC2) from peptide and antibody-conjugated micelles; Figure S7: Determination of apoptotic effects with the mitochondrial membrane potential role of the IC50-loaded DOX molecule-loaded, DOX-molecule-loaded HER2 targeting peptide (LTVSPWY) and monoclonal antibody (Herceptin®) on the SKBR-3 breast cancer cells after 48 hours of incubation as well as method.
