# Supplementary material for: HER2-Specific Peptide (LTVSPWY) and Antibody (Herceptin) Targeted Core Cross-Linked Micelles for Breast Cancer: A Comparative Study
Source: Pharmaceutics. 2023 Feb 22;15(3):733. doi: 10.3390/pharmaceutics15030733 (PMC10053834; doi:10.3390/pharmaceutics15030733)
Supplement: Supplementary file 1 [file pharmaceutics-15-00733-s001.zip › pharmaceutics-2080764-supplementary.pdf]

HER2-specific peptide and antibody (Herceptin) targeted core cross-linked micelles for breast cancer: A comparative study

Nazende Nur Bayram[1], Gizem Tuğçe Ulu[2], Nusaibah Abdulsalam Abdulhadi[2], Seda Gurdap[1], İsmail Alper İşoğlu[1], Yusuf Baran[2], Sevil Dinçer İşoğlu[1]*

[1]Department of Bioengineering, Faculty of Life and Natural Sciences, Abdullah Gül University, Kayseri, Turkey

[2]Molecular Biology and Genetics, Faculty of Science, İzmir Institute of Technology, İzmir, Turkey

sevil.dincer@agu.edu.tr, corresponding author

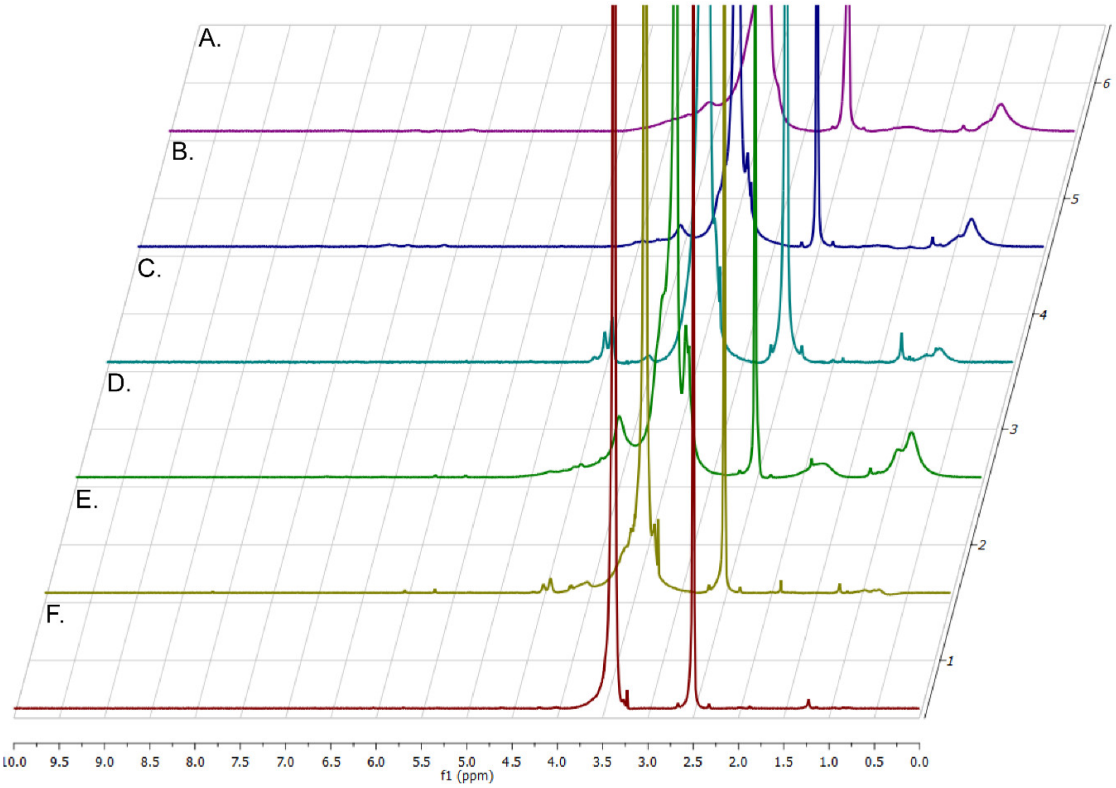

Figure S1. ^{1}H NMR of AC1(A), AC2(B), AC3(C), AC4(D), AC5(E), and CCMs(F) in DMSO.

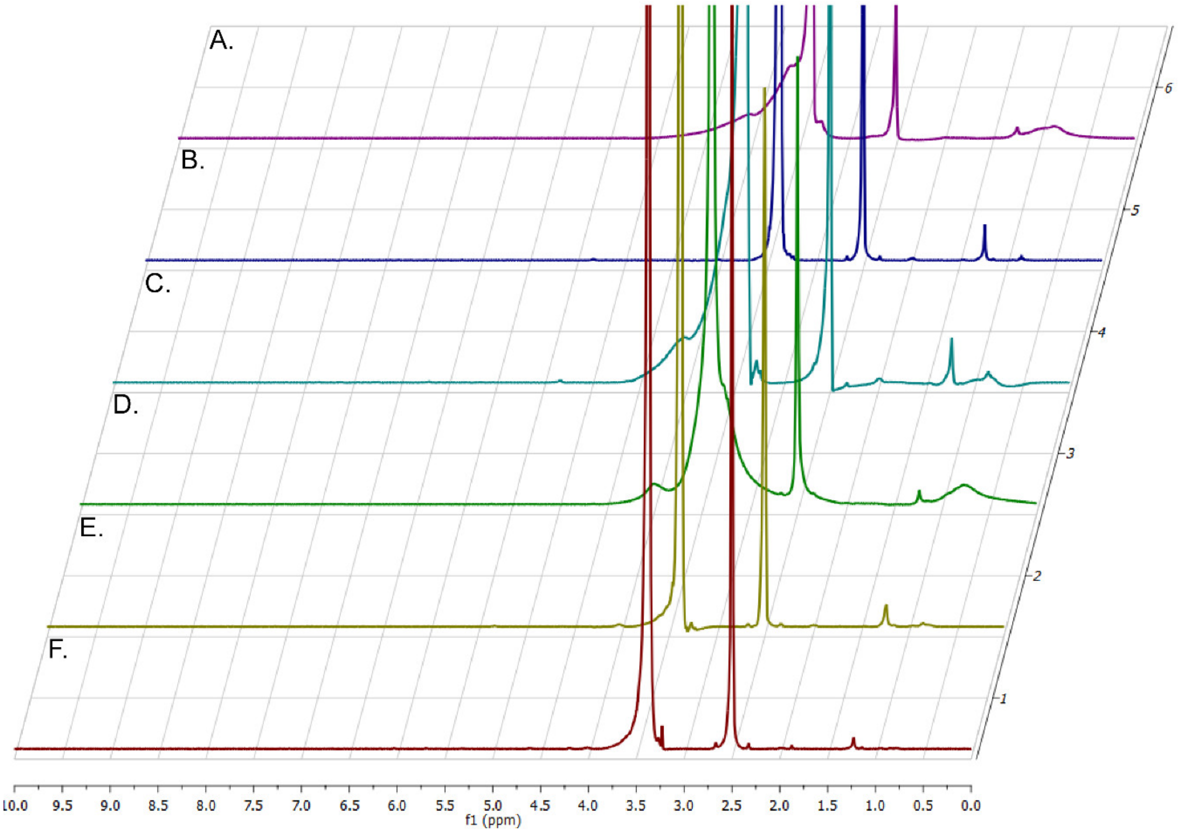

Figure S2. ^{1}H NMR of PC1(A), PC2(B), PC3(C), PC4(D), PC5(E), and CCMs(F) in DMSO.

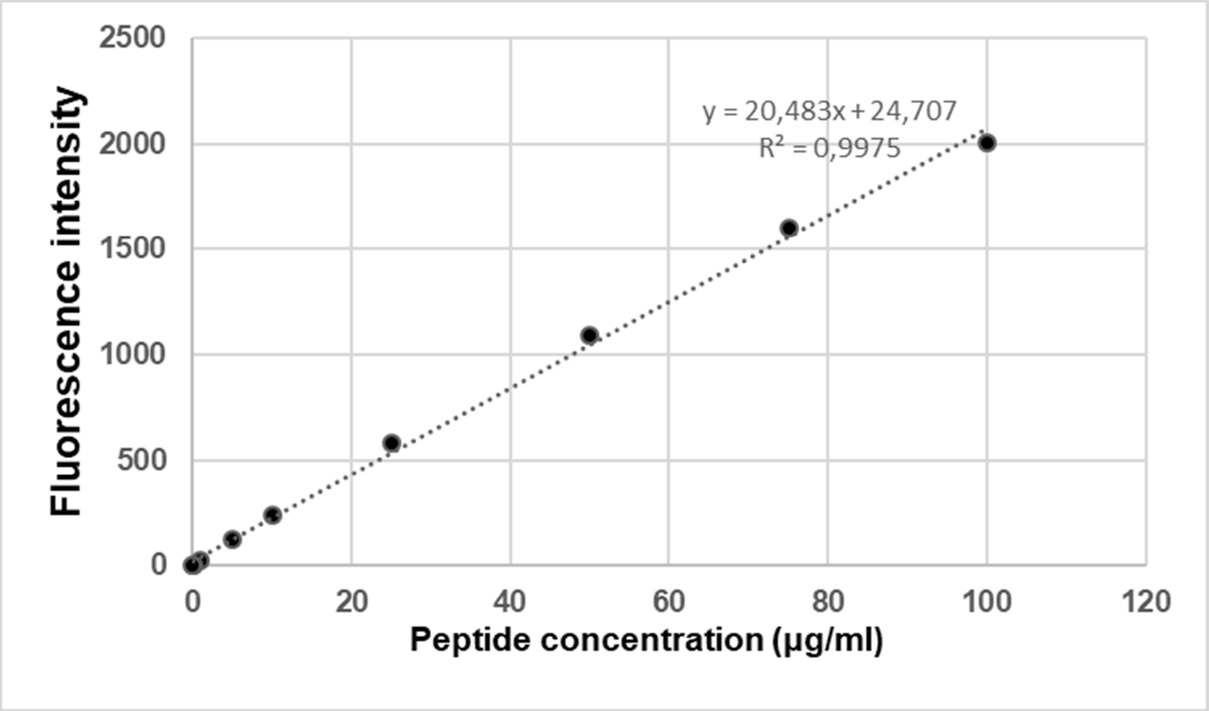

Figure S3. Calibration graph of LTVSPWY peptide in DMSO.

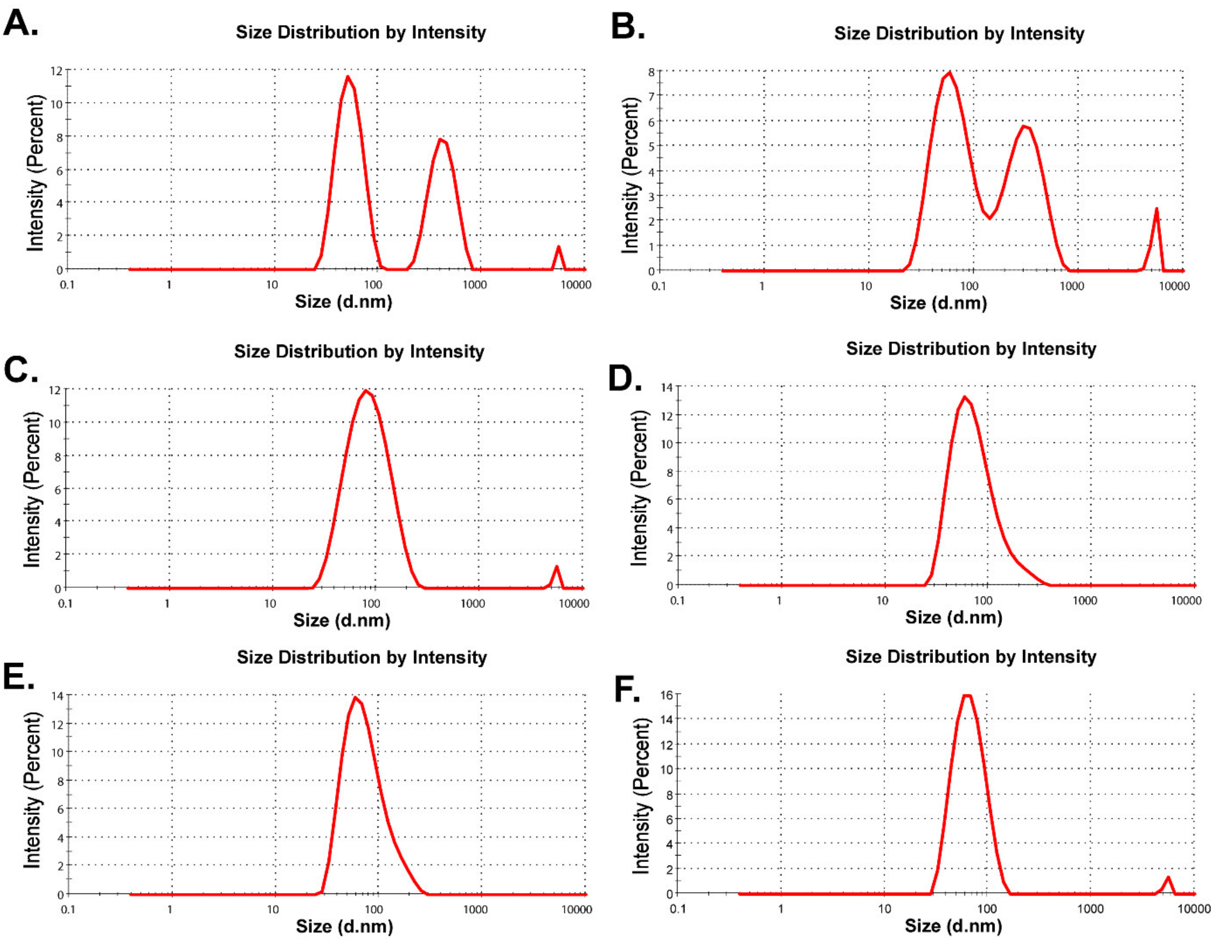

Figure S4. Size distributions of AC1(A) AC2(B) AC3(C) AC4(D) AC5(E) CCMs(F).

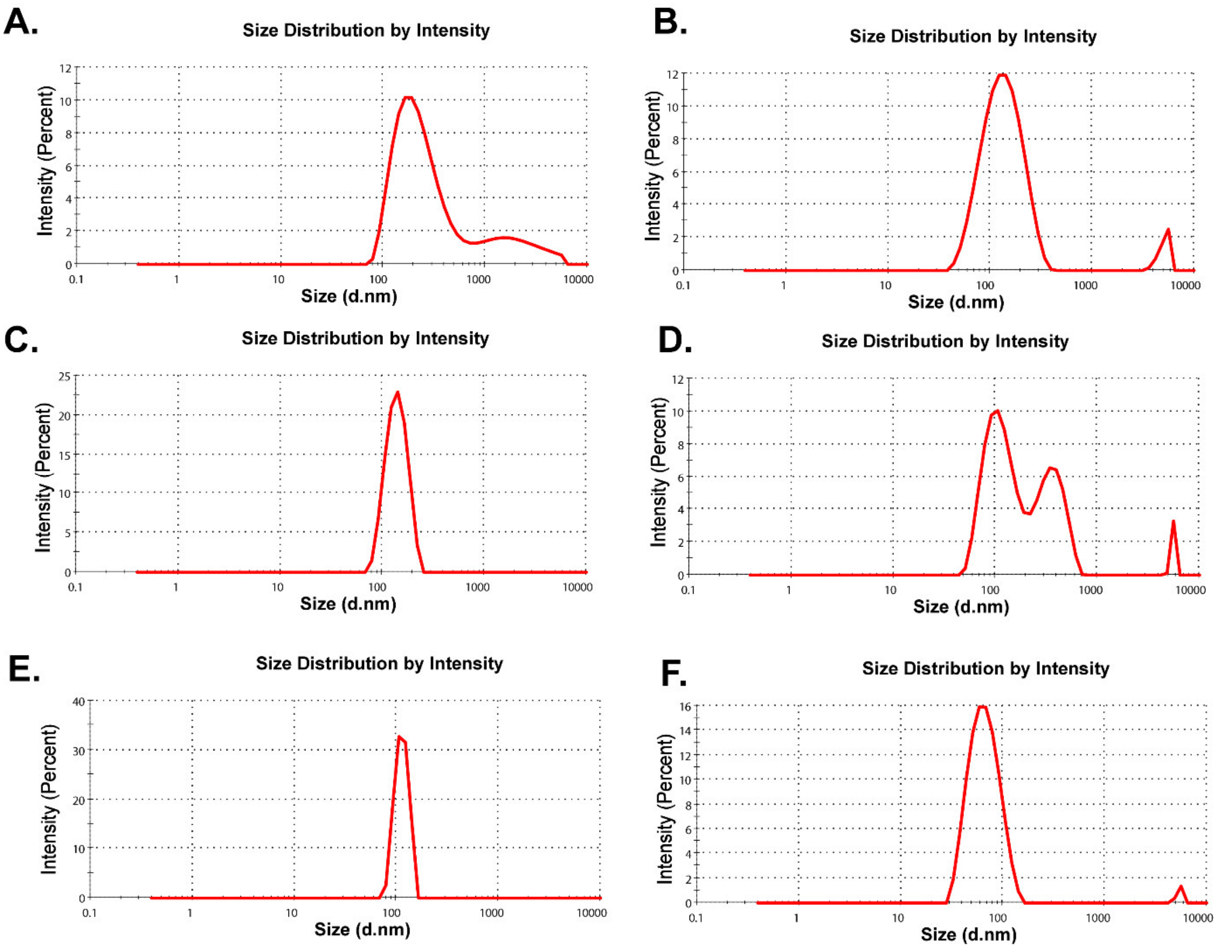

Figure S5. Size distributions of PC1(A) PC2(B) PC3(C) PC4(D) PC5(E) CCMs(F).

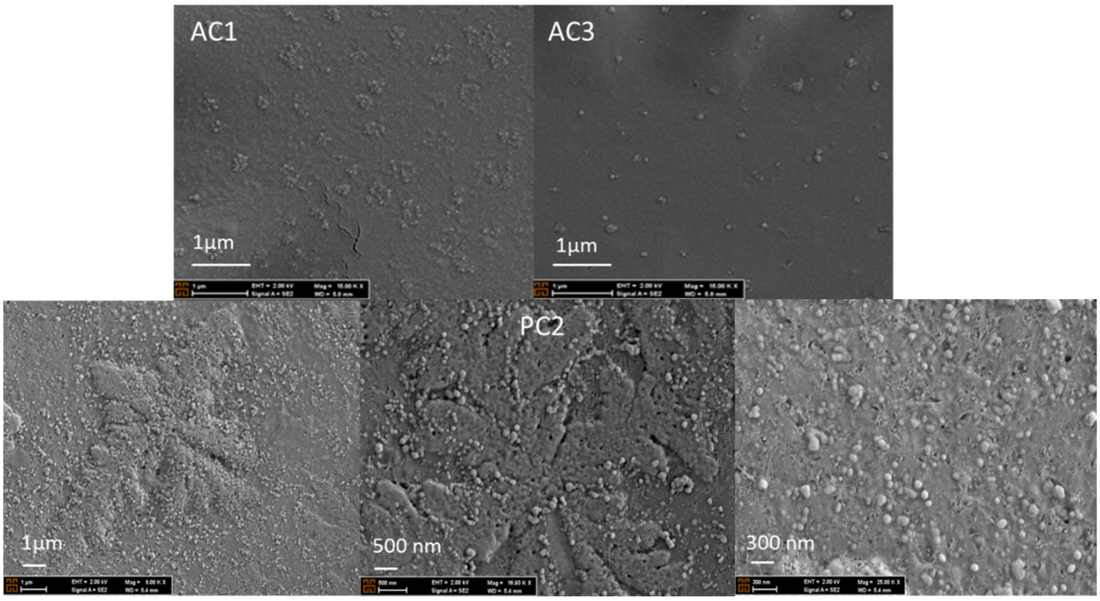

Figure S6. SEM images of selected samples (AC1, AC3, and PC2) from peptide and antibody-conjugated micelles.

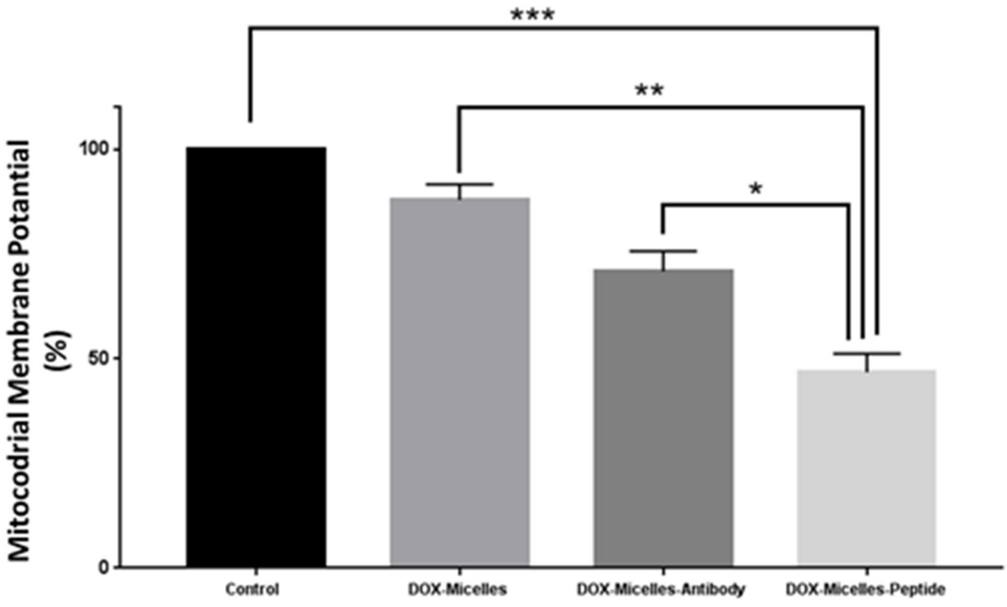

Figure S7. Determination of apoptotic effects with the mitochondrial membrane potential role of the IC50-loaded DOX molecule-loaded, DOX-molecule-loaded HER2 targeting peptide (LTVSPWY) and monoclonal antibody (Herceptin®) on the SKBR-3 breast cancer cells after 48 hours of incubation.

Supplementary methods: Different concentrations of CCMs were applied to the cells into 96 well-plates (Black cell culture plate). After 48 hours of incubation, 10µl JC-1 staining solution (Caymanchem) was added to each well and mixed gently. The plate was incubated in an incubator with 5% CO_2 at 37 °C for 30 minutes. After incubation, the plate was centrifuged, and the supernatant was aspirated. 200µl of Assay buffer was added to each well. JC-1 aggregates for healthy cells and monomers for dead cells were determined for excitation and emission at 535 nm and 595, 485, and 535 nm, respectively. The ratio of JC-1 aggregates to JC-1 monomers was used to determine mitochondrial activity.
